# Supplementary material for: PEG-stabilized coaxial stacking of two-dimensional covalent organic frameworks for enhanced photocatalytic hydrogen evolution
Source: Nat Commun. 2021 Jun 24;12:3934. doi: 10.1038/s41467-021-24179-5 (PMC8225615; doi:10.1038/s41467-021-24179-5)
Supplement: Supplementary file 1 — Supplementary Information [file 41467_2021_24179_MOESM1_ESM.pdf]

# Supplementary Information

## PEG-Stabilized Coaxial Stacking of Two-Dimensional Covalent Organic Frameworks for Enhanced Photocatalytic Hydrogen Evolution

Ting Zhou[1,6], Lei Wang[2,6], Xingye Huang[1], Junjuda Unruangsri[5], Hualei Zhang[1], Rong Wang[1], Qingliang Song[1], Qingyuan Yang[4], Weihua Li[1], Changchun Wang[1], Kaito Takahashi[3*], Hangxun Xu[2*], and Jia Guo[1*]

*[1]State Key Laboratory of Molecular Engineering of Polymers, Department of Macromolecular Science, Fudan University, Shanghai 200433, China.*

*[2]Hefei National Laboratory for Physical Sciences at the Microscale, CAS Key Laboratory of Soft Matter Chemistry, University of Science and Technology of China, Hefei 230026, China.*

*[3]Institute of Atomic and Molecular Sciences Academia Sinica, Taipei 10617, Taiwan.*

*[4]State Key Laboratory of Organic-Inorganic Composites, Beijing University of Chemical Technology, Beijing 100029, China.*

*[5]Research group on Materials for Clean Energy Production STAR, Department of Chemistry, Chulalongkorn University, Bangkok 10330, Thailand.*

*[6]These authors contributed equally: Ting Zhou, Lei Wang.*

**E-mail: guojia@fudan.edu.cn (J. G.), hxu@ustc.edu.cn (H. X.), kt@gate.sinica.edu.tw (K. T.).*

# Section I. Materials and Methods

## 1. Materials

Anhydrous 1,2-dichlorobenzene, anhydrous n-butanol, anhydrous acetonitrile, and chloroplatinic acid hexahydrate were purchased from Aladdin Industrial Corporation. *N,N'*-Dimethylformamide (DMF), tetrahydrofuran (THF), hydrochloric acid (HCl), methanol and acetone were purchased from Shanghai Chemical Regents Company. Sodium hydroxide and ascorbic acid were purchased from Sinopharm Chemical Reagent Co. Ltd. Polyethylene glycol ($M_w$ = 400, 2000 and 20 kDa) was purchased from Shanghai Tuoyang Biotechnology Co. Ltd. 1,3,5-Triformylphloroglucinol, 4,4'-(benzo-2,1,3-thiadiazole-4,7-diyl)dianiline and 4,4'-diamino-*p*-terphenyl were purchased from Jilin Chinese Academy of Sciences-Yanshen technology Co. Ltd. All the chemicals were used without further purification.

## 2. Characterizations

Powder X-ray diffraction (PXRD) patterns were collected at room temperature on an X-ray diffraction spectrometer (Bruker D8 Advance, Germany) with Cu Kα radiation at $\lambda$ = 0.154 nm operating at 40 kV and 40 mA. The simulations of the possible structures were carried out in Accelrys Material Studio (MS) 8.0 software package and further optimized by using the CASTEP module of MS software. The simulated PXRD patterns were determined by the Reflex module. Pawley refinement of the experimental PXRD of BT-COF was conducted to optimize the lattice parameters iteratively until the $R_{wp}$ value converges. Fourier transform infrared (FT-IR) spectra were recorded on Nicolet 6700 (Thermofsher, USA) Fourier transformation infrared spectrometer. Thermogravimetric analyses (TGA) were carried on a Pyris 1 Thermo Gravimetric Analyzer (PE, USA) at a heating rate of 10°C/min from 50°C to 800°C under $N_2$. Diffuse reflectance UV-visible absorption spectra were collected on a Lambda 750 spectrometer (referenced to barium sulphate). Elemental analysis (EA) measurements were analyzed using a vario EL Elemental Analyzer. Inductively coupled plasma atomic emission spectroscopy (ICP-AES) was done on a VARIAN

VISTA RL simultaneous spectrometer (Agilent Technologies, Santa Clara, California, USA) with a CCD-detector. High-resolution transmission electron microscopy images were obtained using a JEOL 2100F microscope operated at 200 kV accelerating voltage. The elemental mappings of C, N, O and S atoms were collected using the same transmission electron microscope (operating at 200 kV) under the ADF STEM mode. $N_2$ adsorption-desorption isotherms were collected by a TriStar II 3020 volumetric adsorption analyzer (Micromeritics, USA) at 77 K. The samples were degassed at 120 °C for 12 h under vacuum before measurement. The surface areas were evaluated using the Brunauer-Emmett-Teller (BET) model applied between P/P0 values of 0.05 and 0.1 for mesoporous COFs. $H_2O$ adsorption measurements were performed at 298K with a sample weight of 20~40 mg and preheated at 120 °C for 12 h. Solid-state CP/MAS $^{13}C$ NMR spectra were recorded on 400WB AVANCE III (Bruker, Switzerland) plus 400 MHz spectrophotometer at 298 K. Differential scanning calorimetry (DSC) was conducted with a TA Q2000 under an atmosphere of $N_2$. Water contact angles were measured using a drop-shape analysis apparatus (Krüss DSA100). The samples were measured using pressed pellets and the contact angles were fitted by an ellipse fitting method. Wide Angle X-ray Scattering (WAXS) measurements were performed on Xeuss 2.0 with a Pilatus 3R 200K-A detector. Copper Kα ($\lambda$ = 1.54 Å) was used as the radiation source. The distance between the sample and the detector is 148.35 mm. The exposing time is 600 s. The scanning degree is ranged from 0.035 to 60 degree. The SEM images were obtained on Zeiss Ultra 55.

## 3. Methods

### 3.1 Synthesis of TP-COF

A Pyrex tube was charged with 1,3,5-triformylphloroglucinol (12.6 mg, 0.06 mmol), 4,4′-diamino-p-terphenyl (23.4 mg, 0.09 mmol), mesitylene (0.5 mL), dioxane (0.5 mL). Pyrrolidine (0.1 mL) was then added into the tube as catalyst. This mixture was homogenized by sonication for 5 minutes and the tube was then flash frozen at 77 K

(liquid $N_2$ bath) and degassed by three freeze-pump-thaw cycles. The tube was sealed and then heated at 120 °C for 3 days. The yellow precipitates were collected by filtration and Soxhlet extracted with tetrahydrofuran for 3 days. After drying at 120 °C under vacuum, the product was obtained a yellow powder (26 mg, 79%).

### 3.2 Synthesis of amorphous poly(TpBT)

A Schleck tube was charged with1,3,5-triformylphloroglucinol (12.6 mg, 0.06 mmol), 4,4′-(benzo-2,1,3-thiadiazole-4,7-diyl)dianiline (28.8 mg, 0.09 mmol), o-dichlorobenzene (5 mL) and aqueous acetic acid (0.5 mL,6 M). This mixture was homogenized by sonication for 5 minutes and the tube was then flash frozen at 77 K (liquid $N_2$ bath) and degassed by three freeze-pump-thaw cycles. This mixture was heated at 120 °C for 3 days. The precipitate was collected by filtration and Soxhelt extracted with tetrahydrofuran for 3 days. After drying at 120 °C under vacuum, the product was obtained a red-colored powder (25 mg, 66%).

### 3.3 Synchrotron radiation photoemission spectroscopy (SRPES) measurement

The measurement was conducted to determine the valence band (VB) positions of both BT-COF and 30%PEG@BT-COF. SRPES experiments were performed at the Photoemission Endstation (BL10B) in the National Synchrotron Radiation Laboratory in Hefei, China. To obtain the secondary electron cutoff, an excitation of 168.8 eV was utilized. The binding energy (BE) was calibrated and referenced to the Ef of a gold foil. The work function (WF) of samples was determined according to the equation $\Phi = hv\text{-}\Delta E$, where $\Delta E$ is the spectrum width, i.e. the energy difference between the secondary electron cutoff and the Fermi level of tested sample. In order to obtain the secondary electron cutoff, a −10 V bias was applied to the sample, which accelerated all the photoelectrons with higher kinetic energy (KE) to overcome the WF of the analyzer.

### 3.4 Photocurrent measurement

The measurements were conducted on a Metrohm Autolab PGSTAT302N potentiostat/galvanostat in a three-electrode cell system under ambient conditions using of a 300 W Xe lamp (Perfect Light PLS-SXE 300). Visible light (λ>420nm) with a power density of 100 mW·cm$^{-2}$ was used as the illumination source. The FTO glasses (1×2 cm$^2$) coated with COFs were as the photoelectrodes. A Pt foil was used as the counter electrode and an Ag/AgCl electrode was used as the reference electrode. The three electrodes were inserted in a quartz cell filled with 0.5 M $Na_2SO_4$ electrolyte. The $Na_2SO_4$ electrolyte was purged with Ar for 1 h prior to the measurements.

### 3.5 The AQE measurement

The apparent quantum efficiency (AQE) was measured under the irradiation of a 300 W Xe lamp with different bandpass filters ($\lambda_0$ ± 20 nm) using the following equation:

$$\eta_{AQE} = \frac{N_e}{N_p} \times 100\% = \frac{2 \times M \times N_A}{\frac{E_{total}}{E_{photon}}} \times 100\% = \frac{2M \times N_A}{\frac{S \times P \times t}{\hbar \times \frac{c}{\lambda}}} \times 100\%$$

$$= \frac{2 \times M \times N_A \times \hbar \times c}{S \times P \times t \times \lambda} \times 100\%$$

Where, $M$ is the amount of $H_2$ molecules (mol), $N_A$ is Avogadro constant (6.022 × 10$^{23}$ mol$^{-1}$), $\hbar$ is the Planck constant (6.626 × 10$^{-34}$ J s), $c$ is the speed of light (3 × 10$^8$ m s$^{-1}$), $S$ is the irradiation area (cm$^2$), $P$ is the intensity of irradiation light (W cm$^{-2}$), $t$ is the photoreaction time (s), $\lambda$ is the wavelength of the monochromatic light (m).

## 4. Calculations

### 4.1 Computational Determination of BT-COF Structure

The eclipsed (AA) and staggered (AB) structures of 2D BT-COF were constructed using the quasi-reactive assembly algorithms (QReaxAA) proposed in our previous work.[1] Molecular mechanics approach was first used to fully optimize the two initial

models using the Smart algorithm implemented in the Forcite module of Materials Studio software, where the DREIDING force field[2] combined with the QEq charge equilibration method[3] were used to describe the bonded and nonbonded interactions between framework atoms. By using the operators of various space groups, the two optimized structures were found to have a symmetry of P-6 and P63/M, respectively. Then, by keeping their symmetries, the two structures were more accurately determined using the Self-Consistent-Charge Density Functional Tight-Binding (SCC-DFTB) method, where both the cell parameters and atomic positions were also allowed to fully relax. Such DFTB method is based on a second-order expansion of the Kohn-Sham total energy in Density Functional Theory (DFT) with respect to charge density fluctuations. All the calculations were accomplished with the aid of the DFTB+ Module implemented in the Materials Studio software, and the Lennard-Jones dispersion correction was employed to describe the involved van der Waals and π-stacking interactions. The Slater-Koster parameter set mio-1-1[4,5] was employed to calculate the interactions of all possible atom-pairs. The convergence thresholds for the optimizations were 0.02 kcal $mol^{-1}$ (energy), 0.1 kcal $mol^{-1}$ $Å^{-1}$ (force), and 0.001 Å (displacement). The self-consistent calculations were performed with the Broyden charge-mixing scheme[6] (an amplitude of 0.2) and the SCC tolerance was taken as $10^{-8}$. The Monkhorst-Pack k-points were set with a separation of 0.04 $Å^{-1}$. Finally, the rational structure was determined via matching the simulated XRD patterns with the experimental one.

**4.2 Electronic Structure Calculations**

Geometry optimization, as well as the calculation of the ionization potential (IP) and electron affinity (EA), was performed using the Coulomb-attenuating method Becke's three parameter hybrid (CAM-B3LYP) functional[7] with the def2SVP basis set.[8] As given in Supplementary Fig. 24, we evaluated three different geometry cutout models, G1-G3, for the BT-COF to confirm the convergence of the energies with respect to size. Following the confirmation that the smallest size cutout can give converged results, we only evaluated the smallest cutout for TP-COF.

For the UV-VIS spectra, we performed calculations using the time dependent (TD) variant[9,10] of the CAM-B3LYP functional with the def2SVP basis set. It has been reported previously that for systems with large charge transfer characteristic in the electronic excitation, TD-B3LYP underestimates the excitation energy, while TD-CAMB3LYP gives the results consistent with experimental values. Therefore, we used TD-CAMB3LYP since the present BT-COF cutout had a very large difference in the electron distribution in the highest occupied molecular orbital (HOMO) and the lowest unoccupied molecular orbital (LUMO) (Supplementary Fig. 25). Furthermore, we optimized the geometries for the first excited state using TD-CAM-B3LYP and evaluated the fluorescence intensity of the transition to the ground electronic state. Solvation was modelled by performing the quantum chemistry calculation using Truhlar's solvation model density (SMD) model.[11] We evaluated the charge transfer in the transition using Ciofini's diagnostics with TD-CAM-B3LYP.[12] For the strong HOMO-LUMO transition, we obtained a charge transfer excitation length of 1.4 Å for BT(G1) while it was 0 for the TP(G1), confirming the importance mentioned above of charge transfer in BT-COF.

To consider the effect of π stacking, we performed the calculation of the π-stacked dimer by taking the optimized geometries for the BT(G1) and stacking it and optimizing the distance between the two planes. Since the binding energy of these species have van der Waals contribution, we used the CAM-B3LYP with Grimm's empirical D3 dispersion correction with Becke-Johnson damping (D3BJ) for these calculations.[13] The solvent effect was modelled using Truhlar's SMD model, similar to all previous calculations. We obtained an optimized stacking distance of 3.7 Å for this BT(D1) geometry, which is slightly larger than the experimental results, but considering that we are calculating for a small cutout and only the dimer structure, we think this is logical. We used SMD-TD-CAM-B3LYP to calculate the UV spectra of this dimer.

Using the ionization potential, electron affinity, and the electronic transition energies, we evaluate the redox potential of these cutout models compared to the requirements for $H_2$ evolution, following the method developed by Zwijnenburg and co-workers.[14,15] We note that Previous studies have used B3LYP functional to perform the geometry optimization, but in the present study, we used CAM-B3LYP. Furthermore, we have also evaluated the effect of using a larger def2TZP basis set toward the UV-VIS spectra and found that the use of a smaller basis set will give results which are to the blue by 0.1 eV.

We optimized the neutral geometry singlet ground electronic state for all the cluster cutout models using CAMB3LYP/defSVP. For the optimization of the cation and anion geometries in the doublet ground electronic state, we used the unrestricted variant of CAMB3LYP. To calculate the free energy's entropic contribution at 298 K, we used harmonic approximation for the vibrational motion. On the other hand, we have ignored the rotational and translational contribution since we use a cluster cutout model that will be immobile in the actual COF geometry. This part is slightly different from the method developed by Zwijnenburg and co-workers,[14,15] but only results in a change of 0.03 eV at most. Taking the free energy differences of the neutral and cation states, we obtained the adiabatic ionization potential (IP). On the other hand, the electron affinity (EA) is obtained from the neural and anion states' energy difference. Using TD-CAMB3LYP, we optimized the geometry on the electronic state corresponding to the strong absorption in the UV spectra given in Supplementary Fig. 24. Taking the free energy difference between the neutral ground state and this electronic excited state, we obtain the adiabatic excitation energy (EX). Using these values, we obtain the ionization potential of the excited state (IP*) by IP-EX, while the electron affinity of the excited state (EA*) is given by EA+EX. We used the IUPAC recommended 4.44 V for the standard hydrogen electrode absolute potential.

We used the same quantum chemistry method, CAMB3LYP-D3/def2SVP with SMD, to estimate the one- and two-hole oxidation potential of ascorbic acid ($H_2A$). In principle, we only need to calculate ascorbate radical (HA) by removing one hydrogen atom, and dehydroascorbic acid (DHA) by removing two hydrogen atoms. However, in the aqueous phase DHA is known to react with water to form the bicyclic diol form (DHAD). Following the study by Tu et al.,[16] we calculated the cluster of ascorbic acid with two water molecules ($H_2A…H_4O_2$), the cluster of ascorbate radical with two water molecules ($HA…H_4O_2$), dehydroascorbic acid with two water molecules ($DHA…H_4O_2$), and the bicyclic diol form of DHA with one water molecule ($DHAD…H_2O$) (Supplementary Fig. 50). Using the free energies based on the harmonic approximation for the vibrational modes of these clusters, we obtain the oxidation potential with respect to the standard hydrogen electrode. To validate the accuracy of the present calculation method to estimate the oxidation potential, we also calculated the oxidation potential for water using the same quantum chemistry methods by calculating $H_2$, $O_2$, and $H_2O$. We find that the present method can give reasonable estimates for the water oxidation potential. As given in Supplementary Table 5, we confirmed that the calculated one- and two-hole oxidation potential for ascorbic acid is negative compared to the calculated water oxidation potential.

**4.3 Computational studies on the binding of PEG with COF**

To evaluate the effect of hydration and polyethylene glycol interaction, we also performed a calculation of a cluster of the BT(G1) with a short PEG model $CH_3CH_2OCH_2CH_3$ (P1), and a longer $CH_3CH_2OCH_2CH_2OCH_2CH_3$ (P2), as well as $H_2O$ molecule. In addition, we evaluated the effect of the P1 and P2 toward the dimer binding energy BT(D1) to clarify if the PEG will strengthen the π-stacking or not.

We calculated the binding energy, in eV, by subtracting the electronic energy of the bound complex from the separated species; therefore, the more positive, the stronger the binding. First, it is important to determine the binding location of the PEG toward the COF. Therefore, we performed a systematic search of the different binding

positions of the P1 model to the BT(G1). Here we considered both Hydrogen bonding to the side as well as on the top of the BT(G1). As given in Supplementary Fig. 19 there are many binding positions for both positions, giving binding energy of 0.4-0.5 eV. This shows that the PEG has many locations to interact with the BT-COF.

The stable binding position from the BT(G1)•••P1 complex was used for the calculation of the binding between P2 and BT(G1). As shown in Supplementary Fig. 20, the binding energy between BT(G1) and P2 has increased slightly compared to that of BT(G1) and P1. Especially, the binding on top has increased greatly with the elongation to model P2, becoming nearly 0.7 eV.

In Supplementary Fig. 21, we present the most stable binding geometries of P1 toward the dimer BT(D1) at three different conditions, sideways, on top, and in between. Compared to the results of Supplementary Fig. 20, the P1 binding energy at the side has increased (0.47 to 0.56 eV). On the other hand, the binding on the surface has decreased slightly (0.5 to 0.47 eV). This latter effect shows that the π-stacking interaction between the COF can weaken the PEG binding on to the surface. In the right column, we present the result where the P1 is placed in between the π-stacked dimer. As seen from the negative energy, this causes the dimer binding between two BT(G1) cutout models to become very weak. Note here that in this calculation, we performed a constraint optimization, where only the distance between the two stacked dimer and the position of the P1 PEG model was optimized. Therefore, we are overestimating the effect of placing the PEG between the COF. However, we think it shows that placing PEG in between the stacked COF is not favorable. The π-stacking distance has increased to 7.65 Å, nearly two times the bare dimer distance of 3.8 Å.

In Supplementary Fig. 22, the results for the P2 binding with BT(D1) are given. We listed two stable geometries for the different binding positions. Compared to the BT(D1)•••P1 given in Supplementary Fig. 21, binding energy has increased, signifying that for the stacked dimer, elongating the PEG chain is favourable for

binding. In Supplementary Table 3, we compare the dimer's binding energy in the presence of the P1 and P2 PEG. As seen from it, the binding of the dimer is about 3.5 eV, and if we have a P2 binding at the side, the binding energy increases to 3.7 eV signifying the strengthening of the $\pi$-stacking with the presence of P2 on the sideways position. The binding of the P1 and P2 on the top of the COF does not affect the binding energy significantly.

Lastly, we believe that the increase in binding energy with the chain length of the PEG model signifies that the long polymers will likely bind to the sideways position stronger than the model provided above. In addition, we only considered the rigid linear structure for the PEG model, but we still found many binding positions between the PEG and BT-COF. Therefore, when the polymer can take bent geometries, many more binding positions can exist, causing stronger binding between the PEG and BT-COF.

**4.4 Theoretical estimation of excited-state lifetime**

Using our cluster cutout model, we estimated the excited state lifetime and the rate for exciton dissociation in the present BT-COF and TP-COF system. Using Einstein's coefficient of spontaneous emission:[17]

$$\mathrm{A}(s^{-1}) = \frac{1}{4\pi\varepsilon_0}\frac{64\pi^4\nu^3}{3hc^3}\mu_{ij} = \frac{2\pi e^2}{\varepsilon_0 m_e c\lambda^2} f_{ij} = 6.67\times10^{13}(nm^2 s^{-1})\frac{f_{ij}}{\lambda^2}$$

where, $\varepsilon_0$ is the vacuum permittivity, $h$ is the Planck's constant, $c$ is the speed of light, ν is the frequency of the emission, $\mu_{ij}$ is the transition dipole moment between states i and j. The second equation is defined using the oscillator strength $f_{ij}$, electron charge $e$, the mass of an electron $m_e$, and the emission light wavelength $\lambda$. We obtain the third equation from the constants' numerical value, where the wavelength is given in nm, and the oscillator strength has no units. Using this equation and the fluorescence peak position and oscillator strength given in Supplementary Table 8, we estimate the spontaneous emission rate to be $3.3\times10^{8}$ $s^{-1}$ and $1.4\times10^{9}$ $s^{-1}$ for BT and TP, respectively. For these COFs, this rough estimate places the lifetime due to the excited state emission to be in ns time scale.

### 4.5 Theoretical estimation of excited state energy transfer rates

For the energy transfer rate or the exciton diffusion rate, we will assume localized excitations and use the formula

$$k_{ET}(s^{-1}) = \frac{2\pi}{\hbar}\left|J_{ij}\right|^2 \int \tilde{\sigma}_{Em}(E)\tilde{\sigma}_{Ab}(E)dE$$

where, $J_{ij}$ is the electron coupling between the two monomer cutouts, and $\tilde{\sigma}_{Em/Ab}(E)$ are the energy dependence of the normalized emission and absorption spectrum. Similar equations have been used for the energy migration in porphyrin-based metal-organic frameworks. [18] The key question here will be how much faster the $k_{ED}$ will be in comparison to the spontaneous emission rate mentioned above. Here we will be modeling the exciton diffusion BT-COF(G1)+BT-COF(G1*)→BT-COF(G1$^*$)+BT-COF(G1), where the electronically excited energy is transferred between neighboring BT monomer units. There are many different ways to obtain the electron coupling, such as using the transition dipole, i.e., the Forster transfer model, or atomic transition densities, or constraint density functional theory.[18,19] Here, we use a dimer model of the G1 cutout model and performed TD-CAMB3LYP calculation to obtain the electronic transition energies. Then half of the energy difference between the 1$^{st}$ and 2$^{nd}$ electronic transitions will be used to estimate the electronic coupling. Here, we note that the 1st and 2nd electronic transitions for the dimer are the symmetric and antisymmetric combination of the monomer excitation. For the in-plane transfer, we will use a linked G1 monomer unit, while for the out-of-plane transition, we will use the stacked dimer (Supplementary Fig. 51). Upon geometry optimization, the stacked dimer complex showed a slight tilting, which is consistent with previous reports on 2D-COF, which mention that AA' stacking is stable, and the perfectly eclipsed form is not the global minima.[20,21] Thus, the two G1 cut out are not perfectly overlapping along the stacking axis. We used the calculated absorption and emission spectra given in Supplementary Fig. 24 to approximate the overlap integral.

As given in Supplementary Table 8, our estimate of the energy transfer rate, $k_{ET}$, of the photoexcited state for the TP- and BT-COF gives values which are several orders faster than the spontaneous emission rate. We can see that the difference in BT and TP-COF's spectral overlap is why we see a difference in the energy transfer rate. An

important aspect of the calculation is that if one looks at the in-plane versus out-of-plane electron coupling, we see that in our model, the out-of-plane values are slightly larger for both COFs. We also evaluated the situation of the perfect overlap. As given in Supplementary Table 8, the coupling becomes twice when the BT-COFs are perfectly stacked, thereby showing how sensitive the electron coupling is toward the stacking geometry. Before ending, we must consider possible errors from our simulation. First, our spectral overlap has been obtained from simplified theoretical spectra rather than experimental results. However, we think the relative shift in peak position for the absorption and emission spectra is reproduced. We do not think the error will be several orders of magnitude, making $k_{ET}$ reach ns time scale.

We do note that recent studies have experimentally shown that the exciton diffusion may not be a step-by-step hopping as assumed here,[19] and domain sizes can affect the exciton diffusion.[22] Therefore, further studies are needed to clarify the transport mechanism fully, but it is beyond the scope of the paper. The methods used here to estimate the rates of emission and energy transfer rely on very simplified models. However, we think the general trend between the TP- and BT-COF can be obtained from these models. On a final note, we mention that recent studies have shown exciton dynamics in ps order, consistent with our estimate given in Table 8.[22,23]

**4.6 Theoretical simulation of polymer conformation.**

Simulations were performed by using dissipative particle dynamics (DPD) method in the canonical ensemble, which is a coarse-gained particle-based mesoscopic simulation technique developed by Hoogerbrugge and Koelman in 1992.[24] The coarse-graining approach is able to capture universal properties of polymers rather than the interactions between specific chemical functional groups. The time evolution of DPD beads with unit mass is governed by Newton's equations (*2*) of motion:[25]

$$\frac{d\mathbf{r}_i}{dt} = \mathbf{v}_i, \frac{d\mathbf{v}_i}{dt} = \mathbf{f}_i$$

$$\mathbf{f}_i = \sum_{j \neq i} \left( \mathbf{F}_{ij}^{\mathrm{C}} + \mathbf{F}_{ij}^{\mathrm{D}} + \mathbf{F}_{ij}^{\mathrm{R}} \right),$$

in which the force is composed of conservative force $\mathbf{F}_{ij}^{\mathrm{C}}$, dissipative force $\mathbf{F}_{ij}^{\mathrm{D}}$, and random force $\mathbf{F}_{ij}^{\mathrm{R}}$. All forces are pairwise-additive, repulsive and short-range with a cutoff at $r_{\mathrm{C}} = 1$.

The conservative force $\mathbf{F}_{ij}^{\mathrm{C}}$ is a soft-repulsive interaction acting along the line of the centers of two particles:

$$\mathbf{F}_{ij}^{C} = \begin{cases} a_{ij}(1 - r_{ij})\hat{\mathbf{r}}_{ij} & (r_{ij} < 1) \\ 0 & (r_{ij} \geqslant 1) \end{cases},$$

where $r_{ij}$ denotes the distance between beads $i$ and $j$,

$$\mathbf{r}_{ij} = \mathbf{r}_i - \mathbf{r}_j,$$

$$r_{ij} = |\mathbf{r}_{ij}|,$$

and $\hat{\mathbf{r}}_{ij}$ represents the unit vector pointing from $j$ to $i$,

$$\hat{\mathbf{r}}_{ij} = \mathbf{r}_{ij}/|\mathbf{r}_{ij}|.$$

The interaction parameter $a_{ij}$ can be estimated from the Flory–Huggins $\chi$-parameter by $a_{ij} \approx 25 + 3.27\chi_{ij}$.[25] $a_{ij}$ was chosen to be 30 between PEG beads, and $a_{ij} = 25$ between the solvent and PEG beads to mimic the solubility of PEG in water.

The dissipative force $\mathbf{F}_{ij}^{D}$ is proportional to the relative velocity and takes the form:

$$\mathbf{F}_{ij}^{\mathrm{D}} = -\gamma w^{\mathrm{D}}(r_{ij})(\hat{\mathbf{r}}_{ij} \cdot \mathbf{v}_{ij})\hat{\mathbf{r}}_{ij},$$

where $\mathbf{v}_{ij} = \mathbf{v}_i - \mathbf{v}_j$ is the relative velocity and $\gamma$ is the friction coefficient.

The random force $\mathbf{F}_{ij}^{\mathrm{R}}$ acts as a heat source to equilibrate the thermal motion and takes the form:

$$\mathbf{F}_{ij}^{\mathrm{R}} = \sigma w^{\mathrm{R}}(r_{ij})\theta_{ij}\hat{\mathbf{r}}_{ij}$$

Where $\sigma$ specifies the noise strength and is set to be 3. $\theta_{ij}(t)$ is a randomly fluctuating variable with zero mean and unit variance, satisfy Gaussian statistics:

$$\langle \theta_{ij}(t) \rangle = 0,$$

$$\langle \theta_{ij}(t)\theta_{kl}(t') \rangle = (\delta_{ik}\delta_{jl} + \delta_{il}\delta_{jk})\delta(t - t').$$

The random force is related to the dissipative force so that they satisfy the fluctuation–dissipation relation:

$$\sigma^2 = 2\gamma k_B T$$

$$w^D(r) = [w^R(r)]^2 = \begin{cases} (1 - r)^2 & (r < 1) \\ 0 & (r \geqslant 1) \end{cases}$$

where $w^D$ and $w^R$ are the $r$-dependent weight functions vanishing at $r > r_C = 1$. Both dissipative force and random force act along the line of centers so that the linear and angular momentums are conservative, ensuring that the simulation is performed in a canonical ensemble.

The beads on the polymeric chain are connected by a harmonic spring potential as:

$$V_{\text{bond}}(r) = \frac{1}{2}k_b(r_b - r_0)^2$$

Where $r_0$ is the equilibrium length and was set to be 0.75, $r_b$ is the distance between connected beads, $k_b$ is the spring constant and was chosen to be 30.0.

The PEG chain was modeled by DPD beads sequentially linked by harmonic bond, and it was confined in a nanotube, while the water solvent was modeled by single-bead molecules.

The constraint of the nanotube to the PEG and solvents was implemented by the hard-repulsive Lennard-Jones potential:

$$V_{LJ}(r) = \begin{cases} 4\varepsilon\left[\left(\frac{\sigma}{r}\right)^6 - \left(\frac{\sigma}{r}\right)^{12}\right] & r < r_{cut} \\ 0 & r \geq r_{cut} \end{cases}$$

Where $\varepsilon$ is the depth of the potential at its minimum and was set to be 1.0, $r$ is the distance between PEG/solvent beads and the pore wall, and $r_{cut} = 1.1225$ is the cutoff of the Lennard-Jones force.

The hydrogen bonding attractive interaction between PEG and nanotube was qualitatively mimicked by a Morse interaction as:

$$V_{morse}(r) = \begin{cases} D_0\left[exp\left(-2\alpha(r-r_0)\right) - 2exp\left(-\alpha(r-r_0)\right)\right] & r < r_{cut} \\ 0 & r \geq r_{cut} \end{cases}$$

Where $D_0$ is the depth of the potential at its minimum and was set to be 1.0, $\alpha$ controls the width of the potential well and was set to be 3.0, $r_0$ is the position of the minimum and was set to be 1.0, and $r_{cut}$ is the cutoff of the Morse force and was set to be 3.0.

The number density of beads was fixed as 3 by setting total number of polymeric and solvent beads as well as the sizes of the simulation box. The system temperature was maintained at $T^* = 1.0$. The radius of interaction between molecules and the particle mass were set to unity, leading to the reduced unit of dimensionless time of the system as $\tau = R_{\mathrm{c}}\sqrt{m/k_{\mathrm{B}}T}$. The Velocity-Verlet integration scheme was used to integrate the equations of motion. The time-step was set to be $0.001\tau$ to achieve a balance between simulation stability and performance.

All the simulated systems were randomly positioned in the simulation box and were simulated $1 \times 10^3\, \tau$ to generate a disordered state. Simulations of $1 \times 10^4 \tau$ were performed to anneal the system toward the equilibrium morphology.

The initial configuration was generated by the open-source package GALAMOST (version 4.0.1), developed and maintained by Zhu.[26] Simulations were performed using HOOMD-Blue,[27] v.2.9.3, a free and open-source code developed and maintained at the University of Michigan.

The modeled polymer chain was placed in a cylindrical tube with periodic condition in the z direction and no space constraint solvent state, respectively. The radius of

the tube and the number of the beads in the polymeric chain was varied to investigate the confinement effect of the nanotube on the conformation of the PEG chain. As a comparison, the conformation of a single PEG chain in the bulk solvent is also studied in our simulations. Specifically, we calculate the mean square radius of gyration of the PEG chain confined in nanotubes with various diameters as well as that of the PEG chain in the bulk solvent. The mean square radius of gyration is calculated by the following expression:

$$< R_g^2 >= \frac{1}{N^2}\sum_{i=1}^{N}\sum_{j=1}^{N} < \left(\vec{R}_i - \vec{R}_j\right)^2 >$$

Where $N$ is the number of beads in the PEG chain, $\vec{R}_i$ and $\vec{R}_j$ is the position vector of bead $i$ and $j$. For every point in Supplementary Fig. 42d, 200 samples were sampled to calculate the mean of the radius of gyration. The elongated conformation of the PEG chain in small nanotubes is clearly evidenced by our simulation results.

## Section II. Supplementary Figures and Tables

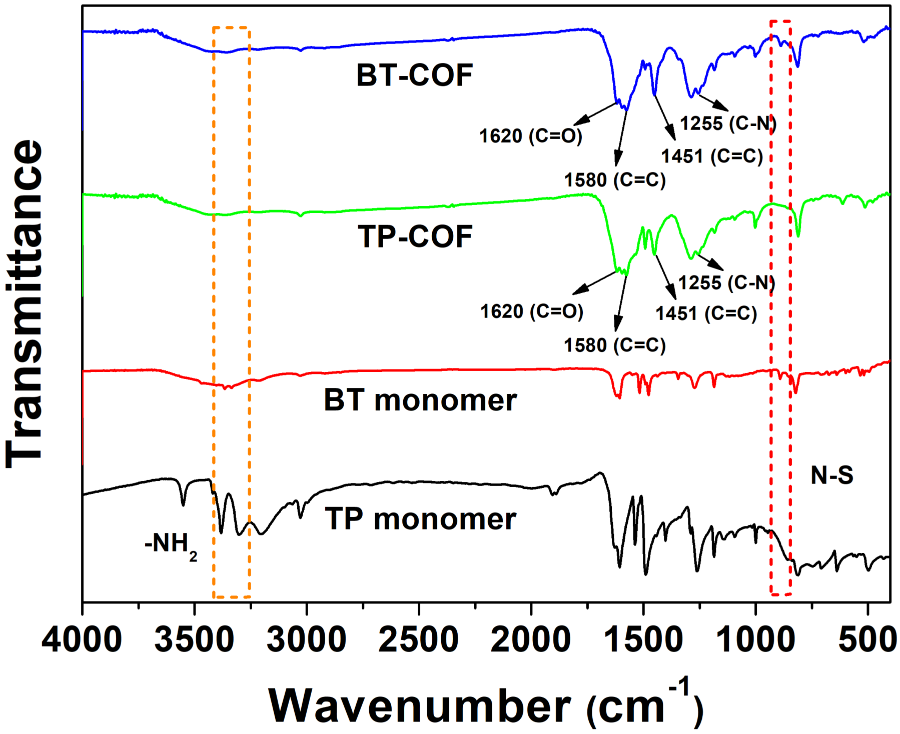


**Fig. 1** FT-IR spectra of BT-COF, TP-COF, BT monomer and TP monomer. The appearance of the two peaks at 1451 and 1255 $cm^{-1}$ corresponding to the aromatic C=C and newly formed C−N bonds, confirms the formation of COFs in the keto-enamine form with the expected linkages. The characteristic stretching band of the C=O bond at 1620 $cm^{-1}$ is observed both in BT-COF and TP-COF.

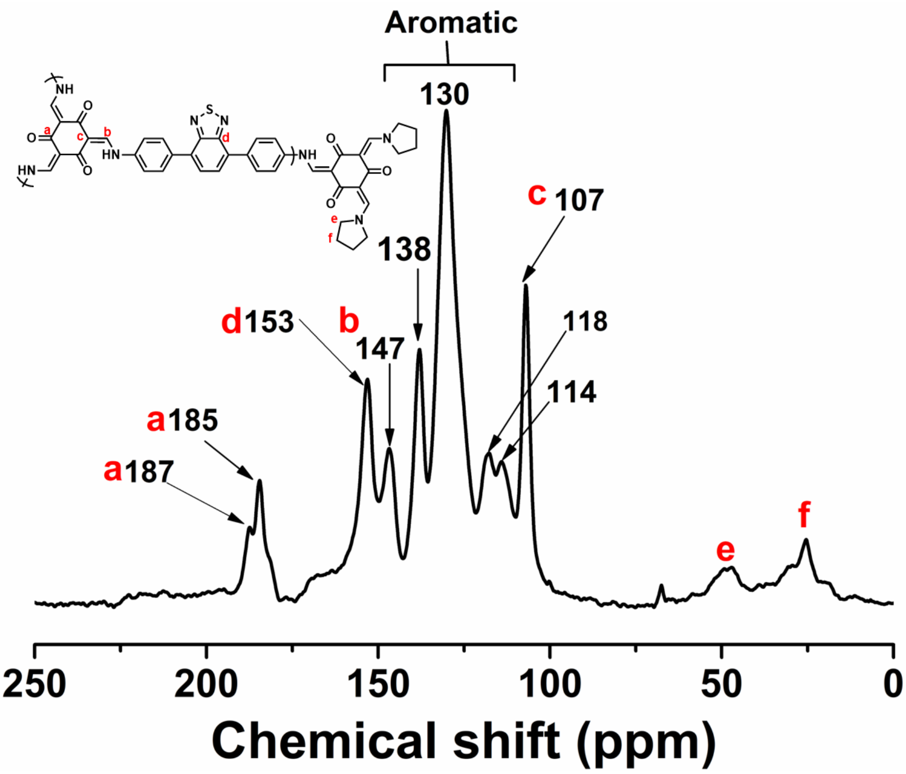


**Fig. 2** Solid-state $^{13}C$ CP-MAS NMR spectrum of BT-COF.

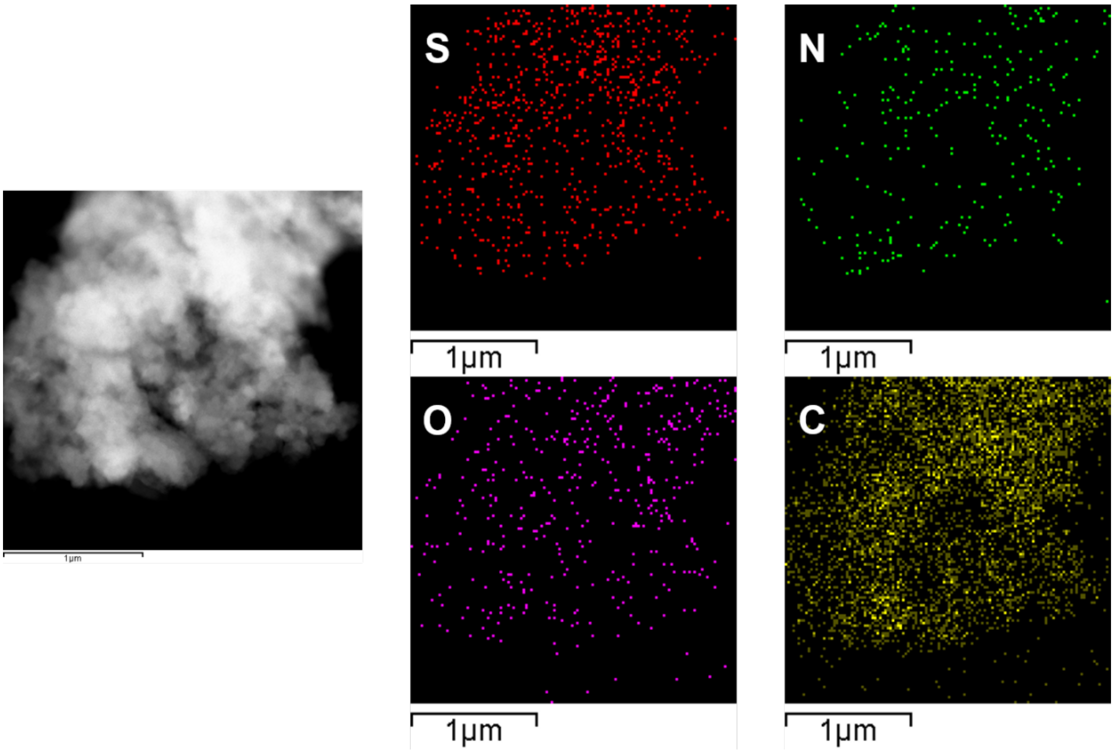


**Fig. 3** FE SEM image of the BT-COF and its corresponding elemental mappings of S (red), N (green), O (purple) and C (yellow) atoms.

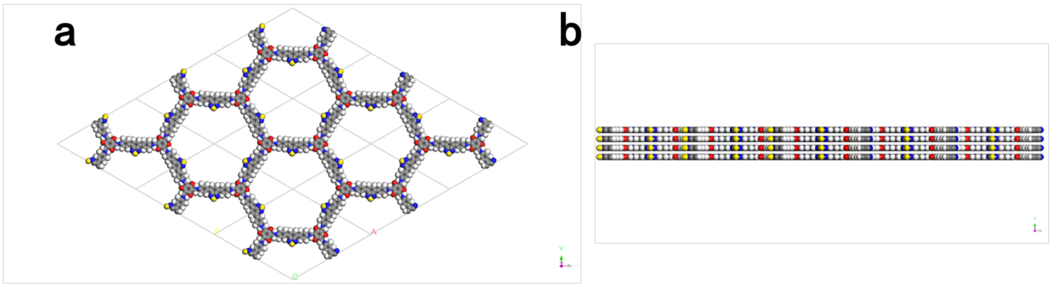


Space group: P6

a = b = 38.0430 Å, c = 3.46850 Å, α = β = 90°, γ = 120°, V = 4347.3 Å$^3$

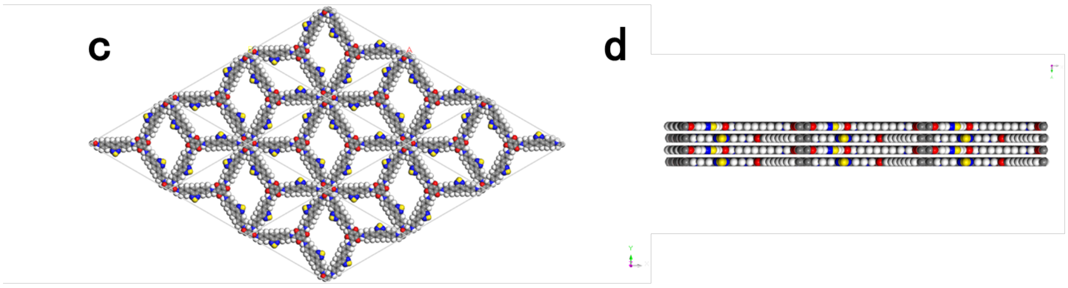


Space group: P63/M

a = b = 38.0863 Å, c = 6.09630 Å, α = β = 90°, γ = 120°, V = 7640.9 Å$^3$

**Fig. 4** The computationally determined structures of BT-COF. (a) The theoretical structure of BT-COF with eclipsed (AA) stacking arrangement. (b) Side view of the BT-COF structure with eclipsed stacking arrangement. (c) The theoretical structure of the BT-COF with staggered (AB) stacking arrangement. (d) Side view of the BT-COF structure with staggered stacking arrangement.

**Table 1.** The atomistic coordinates of BT-COF with AA and AB stacking generated by calculations.

| BT-COF<br>AA Stacking<br>Space group symmetry: P6<br>a = b = 38.0430 Å; c = 3.46850 Å<br>α = β = 90°, γ = 120° | | | | |
|---|---|---|---|---|
| C1 | C | 0.30992 | 0.13701 | 0 |
| C2 | C | 0.3529 | 0.15649 | 0 |
| C3 | C | 0.3786 | 0.19843 | 0 |
| C4 | C | 0.35781 | 0.22171 | 0 |
| C5 | C | 0.31289 | 0.20136 | 0 |
| C6 | C | 0.28799 | 0.15737 | 0 |
| C7 | C | 0.24305 | 0.13527 | 0 |
| C8 | C | 0.42332 | 0.21697 | 0 |
| C9 | C | 0.44194 | 0.19274 | 0 |
| C10 | C | 0.48392 | 0.20964 | 0 |
| C11 | C | 0.51002 | 0.25217 | 0 |
| C12 | C | 0.49204 | 0.27683 | 0 |
| C13 | C | 0.44996 | 0.25948 | 0 |
| C14 | C | 0.22048 | 0.15564 | 0 |
| C15 | C | 0.17828 | 0.13477 | 0 |
| C16 | C | 0.15583 | 0.09193 | 0 |
| C17 | C | 0.17772 | 0.07099 | 0 |
| C18 | C | 0.22006 | 0.09232 | 0 |
| N19 | N | 0.11353 | 0.0731 | 0 |
| N20 | N | 0.5519 | 0.26738 | 0 |
| C21 | C | 0.08609 | 0.03292 | 0 |
| C22 | C | 0.04429 | 0.01775 | 0 |
| C23 | C | 0.58177 | 0.30675 | 0 |
| C24 | C | 0.62291 | 0.3188 | 0 |
| N25 | N | 0.37711 | 0.26193 | 0 |
| S26 | S | 0.33947 | 0.27807 | 0 |
| N27 | N | 0.29764 | 0.22597 | 0 |
| C28 | C | 0.02693 | 0.04486 | 0 |
| C29 | C | 0.637 | 0.289 | 0 |
| H30 | H | 0.29367 | 0.10367 | 0 |
| H31 | H | 0.36588 | 0.13639 | 0 |
| H32 | H | 0.42336 | 0.15949 | 0 |
| H33 | H | 0.49712 | 0.18972 | 0 |
| H34 | H | 0.51113 | 0.31003 | 0 |
| H35 | H | 0.4372 | 0.27981 | 0 |

| | | | | |
|---|---|---|---|---|
| H36 | H | 0.2365 | 0.18897 | 0 |
| H37 | H | 0.16195 | 0.1518 | 0 |
| H38 | H | 0.16154 | 0.03766 | 0 |
| H39 | H | 0.23544 | 0.07439 | 0 |
| H40 | H | 0.09742 | 0.01128 | 0 |
| H41 | H | 0.57304 | 0.3304 | 0 |
| O42 | O | 0.04914 | 0.08249 | 0 |
| O43 | O | 0.61244 | 0.2518 | 0 |
| H44 | H | 0.10033 | 0.09168 | 0 |
| H45 | H | 0.56257 | 0.24667 | 0 |

| BT-COF AB Stacking<br>Space group symmetry: P63/m<br>a = b = 38.0863 Å; c = 6.09630 Å<br>α = β = 90°, γ = 120° | | | | |
|---|---|---|---|---|
| C1 | C | 0.30995 | 0.13643 | 0.25 |
| C2 | C | 0.35291 | 0.15589 | 0.25 |
| C3 | C | 0.37862 | 0.19778 | 0.25 |
| C4 | C | 0.35783 | 0.22102 | 0.25 |
| C5 | C | 0.31293 | 0.20069 | 0.25 |
| C6 | C | 0.28802 | 0.15674 | 0.25 |
| C7 | C | 0.24311 | 0.13469 | 0.25 |
| C8 | C | 0.42332 | 0.21634 | 0.25 |
| C9 | C | 0.442 | 0.1922 | 0.25 |
| C10 | C | 0.48395 | 0.20914 | 0.25 |
| C11 | C | 0.51003 | 0.25162 | 0.25 |
| C12 | C | 0.49199 | 0.27619 | 0.25 |
| C13 | C | 0.44994 | 0.25881 | 0.25 |
| C14 | C | 0.22056 | 0.15504 | 0.25 |
| C15 | C | 0.17839 | 0.13423 | 0.25 |
| C16 | C | 0.15584 | 0.09142 | 0.25 |
| C17 | C | 0.17774 | 0.07053 | 0.25 |
| C18 | C | 0.22004 | 0.09177 | 0.25 |
| N19 | N | 0.11361 | 0.07274 | 0.25 |
| N20 | N | 0.55189 | 0.26688 | 0.25 |
| C21 | C | 0.08605 | 0.03278 | 0.25 |
| C22 | C | 0.0442 | 0.01768 | 0.25 |
| C23 | C | 0.58168 | 0.30619 | 0.25 |
| C24 | C | 0.62288 | 0.31851 | 0.25 |
| N25 | N | 0.3771 | 0.26118 | 0.25 |
| S26 | S | 0.33952 | 0.27733 | 0.25 |
| N27 | N | 0.29772 | 0.22529 | 0.25 |

| | | | | |
|---|---|---|---|---|
| C28 | C | 0.02687 | 0.04468 | 0.25 |
| C29 | C | 0.63731 | 0.28905 | 0.25 |
| H30 | H | 0.29373 | 0.10314 | 0.25 |
| H31 | H | 0.36585 | 0.1358 | 0.25 |
| H32 | H | 0.42347 | 0.15898 | 0.25 |
| H33 | H | 0.49712 | 0.18922 | 0.25 |
| H34 | H | 0.51099 | 0.30935 | 0.25 |
| H35 | H | 0.43717 | 0.27909 | 0.25 |
| H36 | H | 0.2366 | 0.18835 | 0.25 |
| H37 | H | 0.16217 | 0.15134 | 0.25 |
| H38 | H | 0.16157 | 0.03724 | 0.25 |
| H39 | H | 0.23538 | 0.07385 | 0.25 |
| H40 | H | 0.09726 | 0.01107 | 0.25 |
| H41 | H | 0.57283 | 0.32971 | 0.25 |
| O42 | O | 0.0492 | 0.08249 | 0.25 |
| O43 | O | 0.61292 | 0.25178 | 0.25 |
| H44 | H | 0.10072 | 0.09149 | 0.25 |
| H45 | H | 0.56248 | 0.24617 | 0.25 |

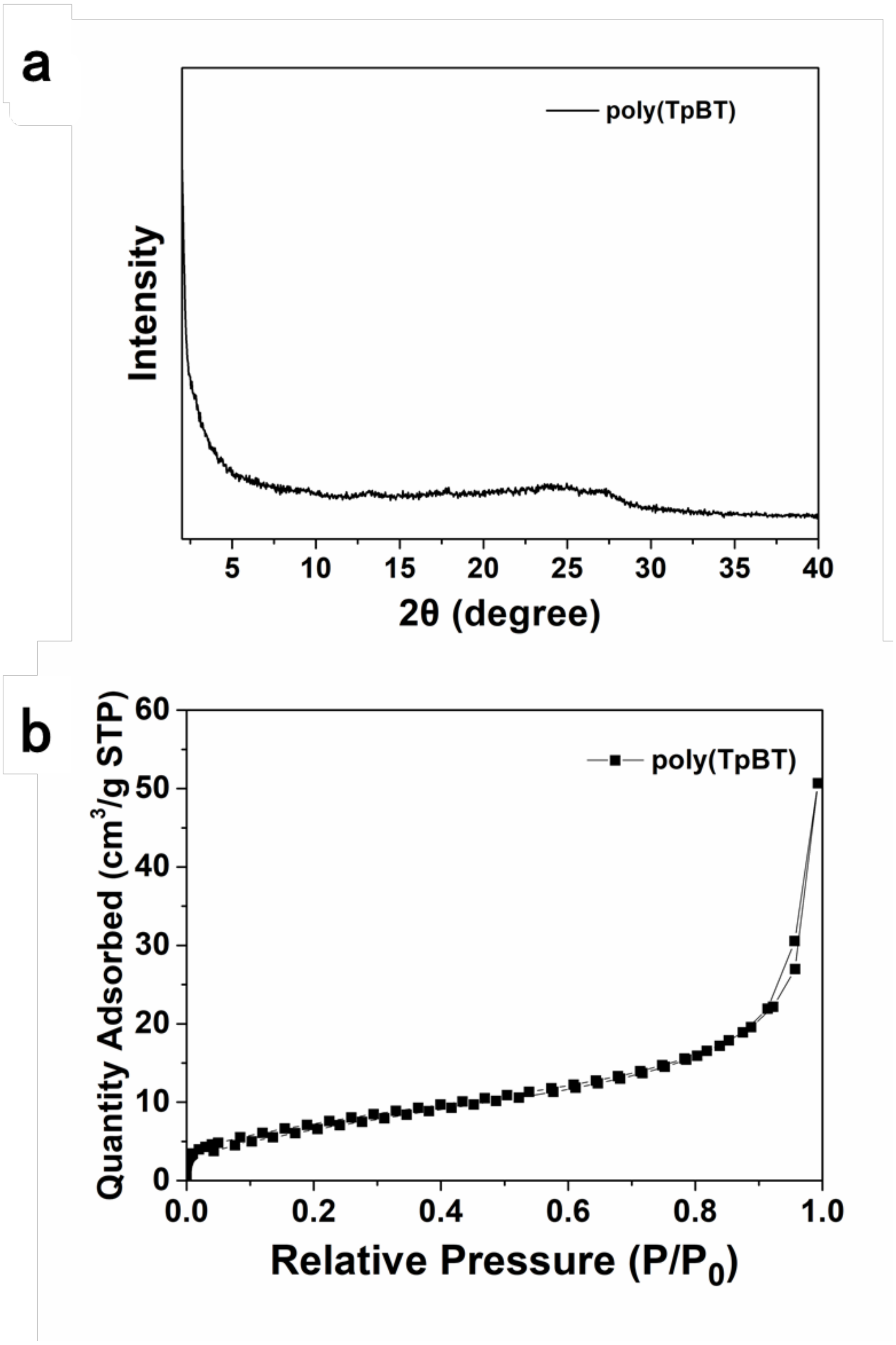


**Fig. 5** PXRD pattern (a) and $N_2$ sorption isotherms (b) of the amorphous poly(TpBT).

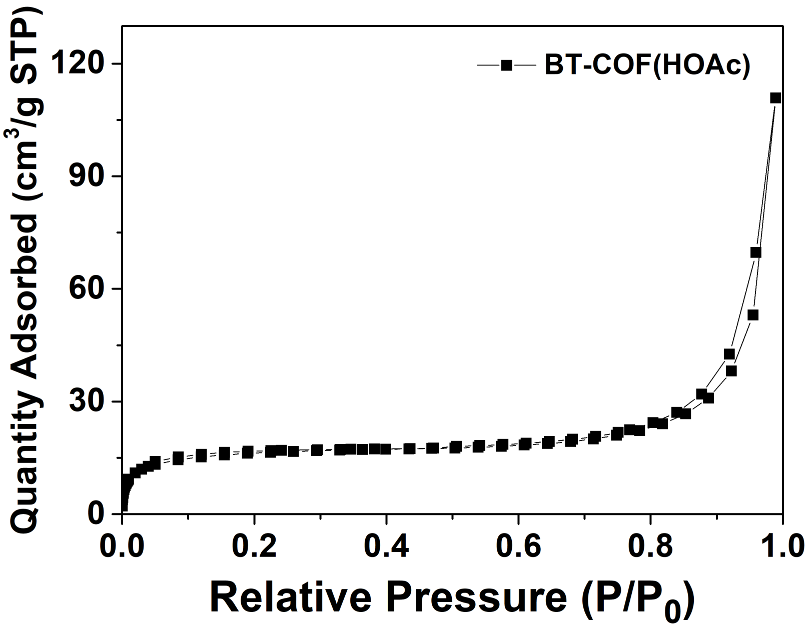


**Fig. 6** $N_2$ sorption isotherms of the low crystalline BT-COF synthesized using 6M HOAc aqueous solution as a catalyst.

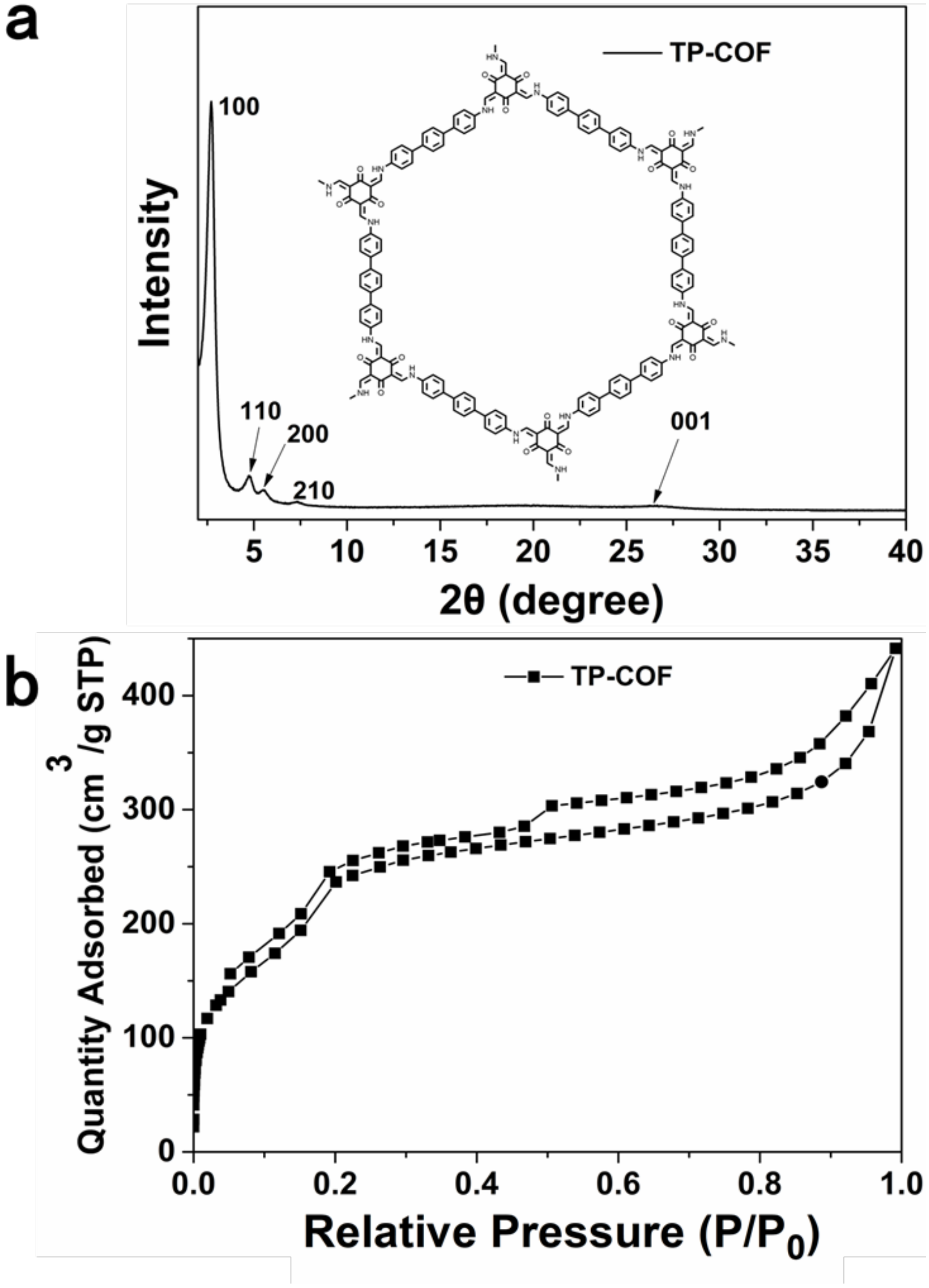


**Fig. 7** PXRD pattern (a) and $N_2$ sorption isotherms (b) of the TP-COF synthesized with pyrrolidine as catalyst under otherwise identical conditions.

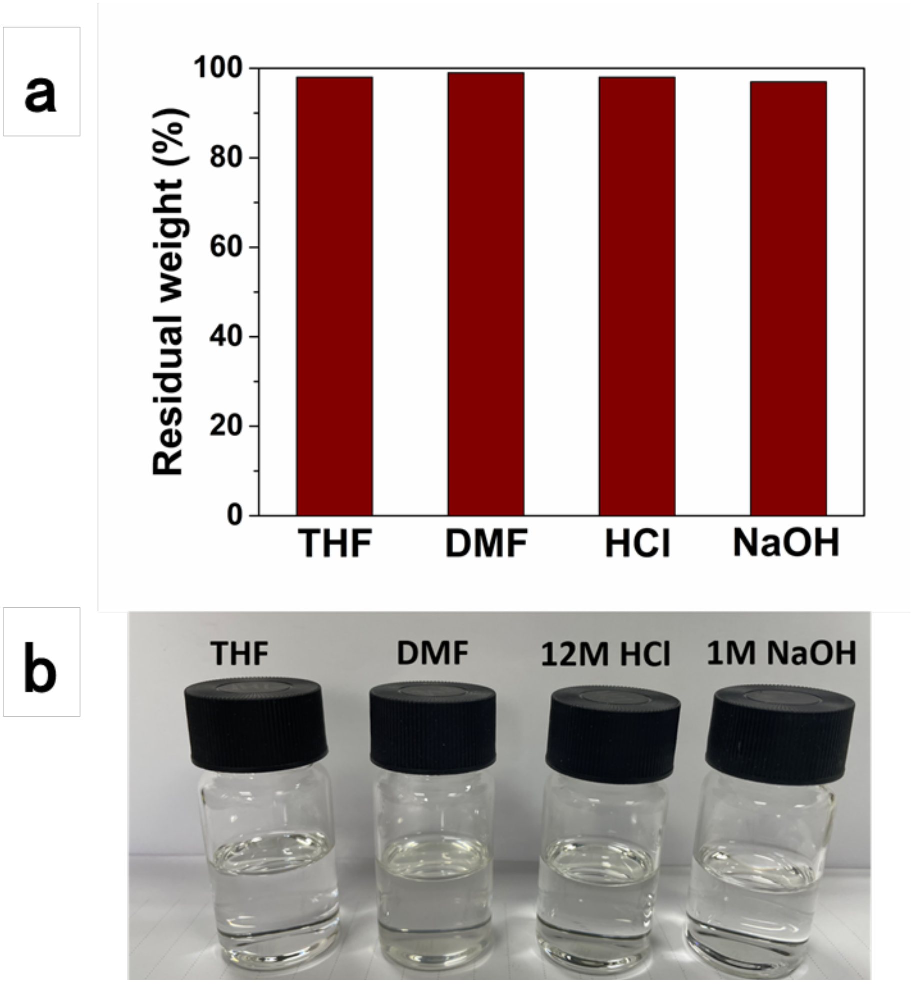


**Fig. 8** The remaining weights (a) and supernatants photographs (b) for the treated BT-COF with different solvents including THF, DMF, HCl (12 M) and NaOH aq. solution (1M), respectively.

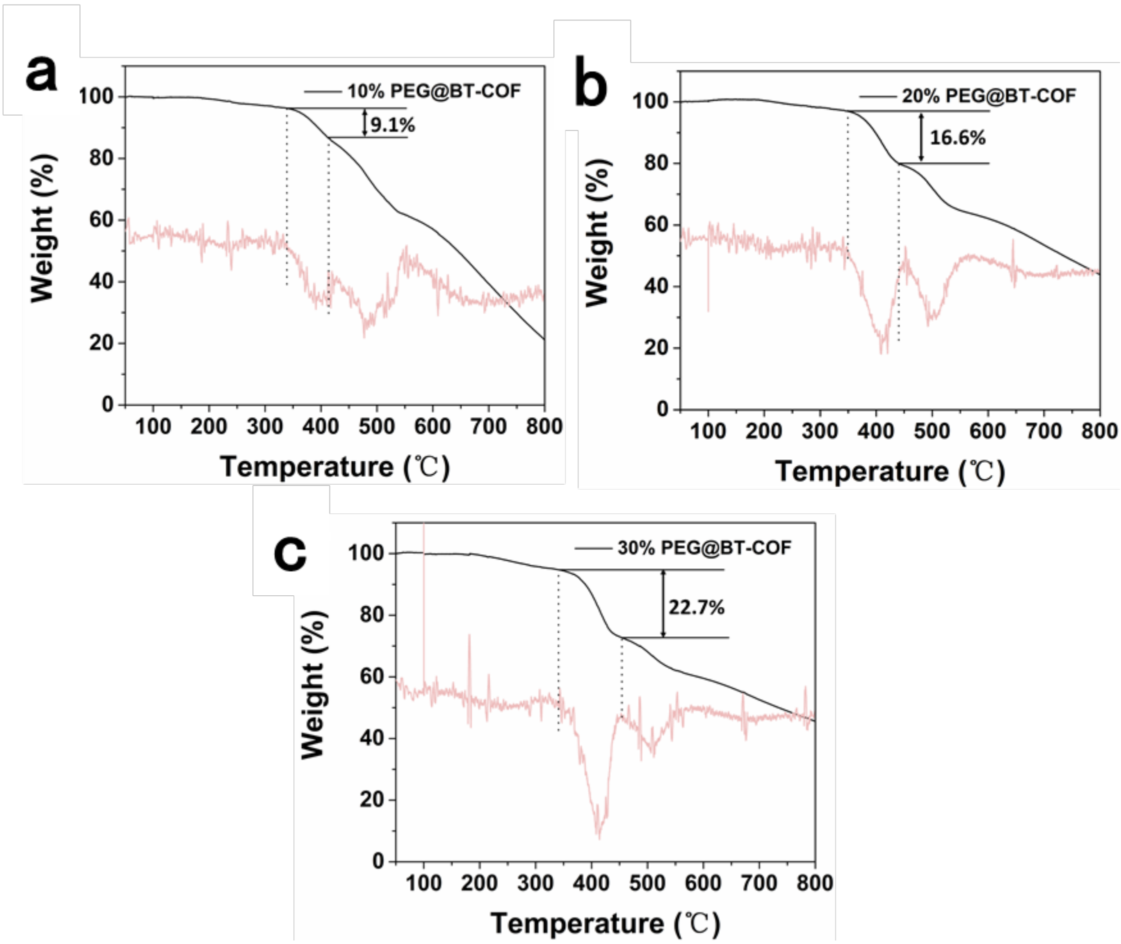


**Fig. 9** TGA profiles of 10%PEG@BT-COF (a), 20%PEG@BT-COF (b) and 30%PEG@BT-COF (c), respectively. As the temperature range for the PEG thermal decomposition is determined in the range from 350ºC to 450ºC, the weight percentages of the loaded PEG relative to the total mass of PEG@BT-COF are found at 9.1%, 16.6% and 22.7%, respectively. They are almost the same with the feeding contents, i.e. 9.09%, 16.67% and 23.08%, calculated from the 10%PEG@BT-COF, 20%PEG@BT-COF and 30%PEG@BT-COF, respectively.

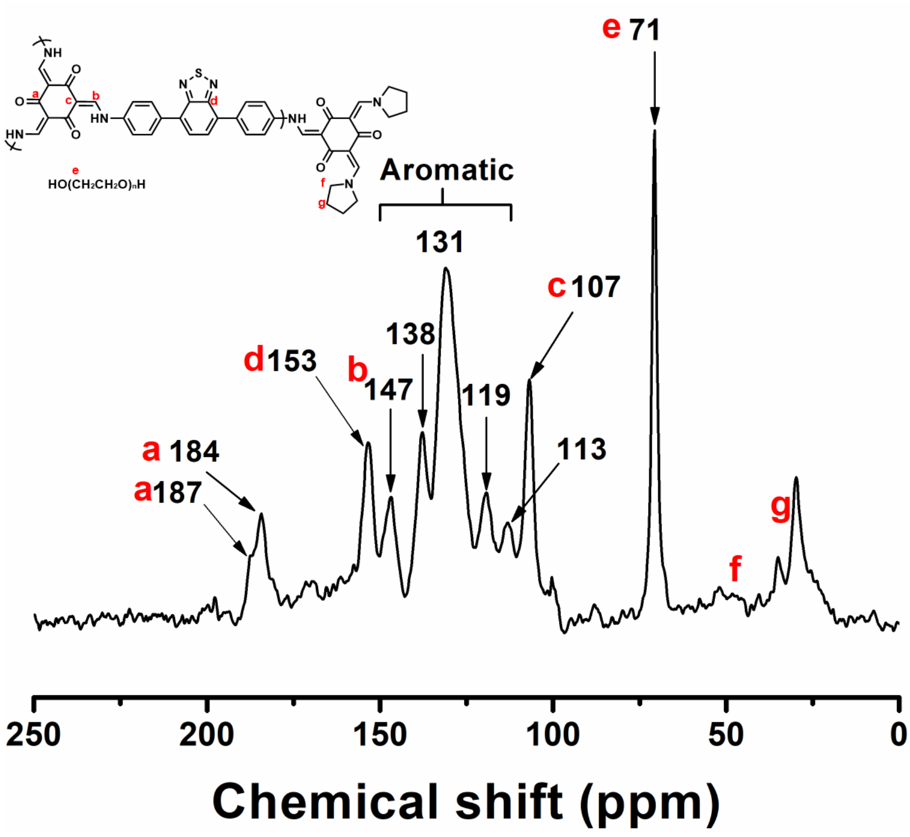


**Fig. 10** Solid-state $^{13}$C CP/MAS NMR spectra of PEG@BT-COF.

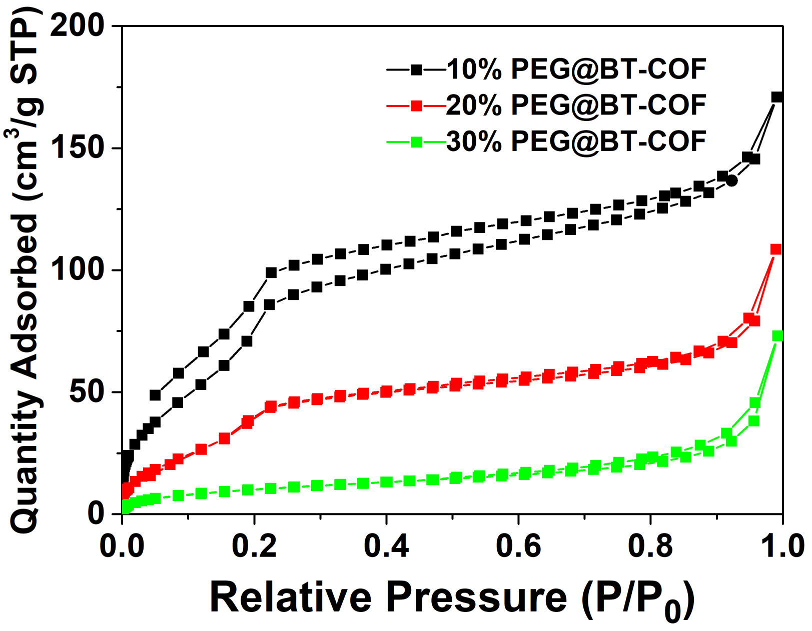


**Fig. 11** $N_2$ sorption isotherms of 10%PEG@BT-COF, 20%PEG@BT-COF and 30%PEG@BT-COF, respectively.

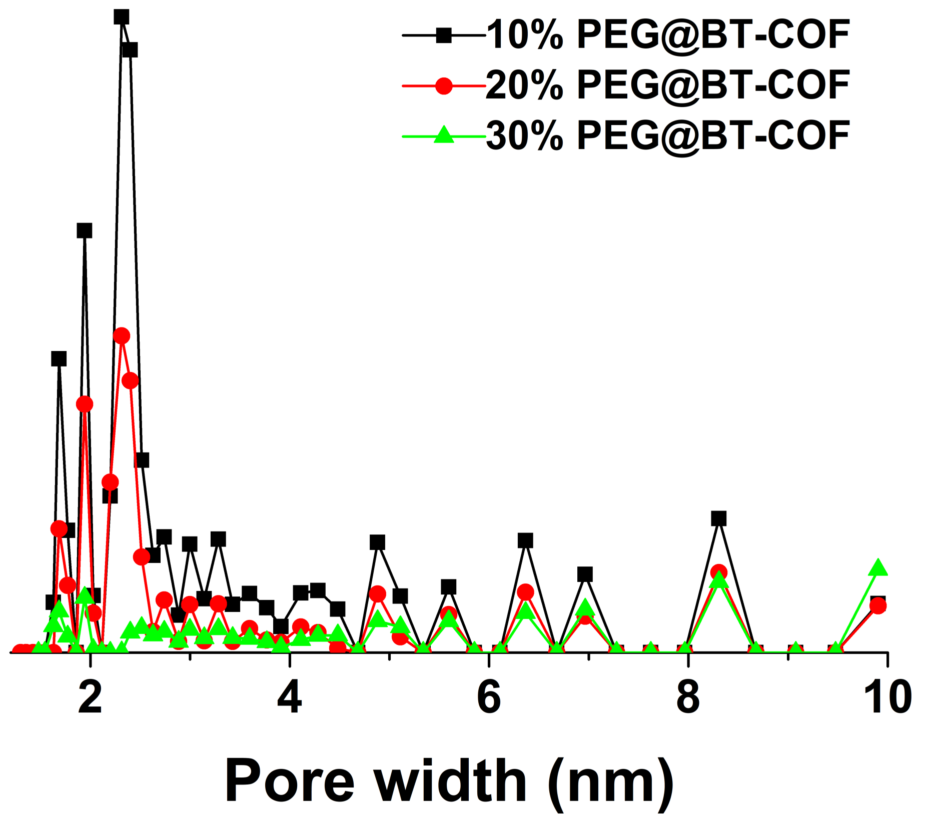


**Fig. 12** Pore-size distributions of 10%PEG@BT-COF, 20%PEG@BT-COF and 30%PEG@BT-COF, respectively.

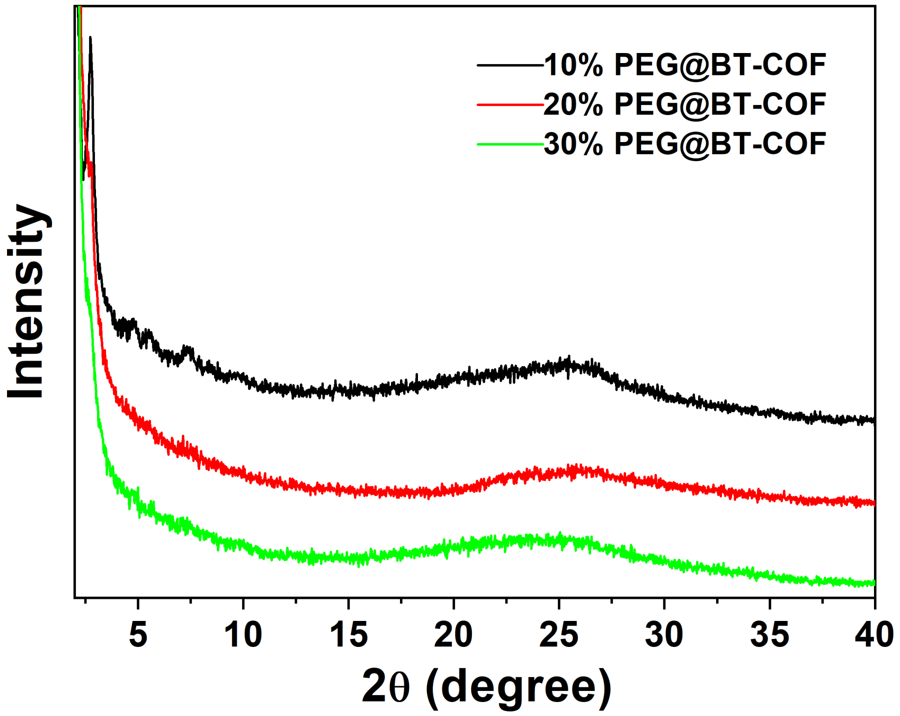


**Fig. 13** PXRD patterns of 10%PEG@BT-COF, 20%PEG@BT-COF and 30%PEG@BT-COF, respectively.

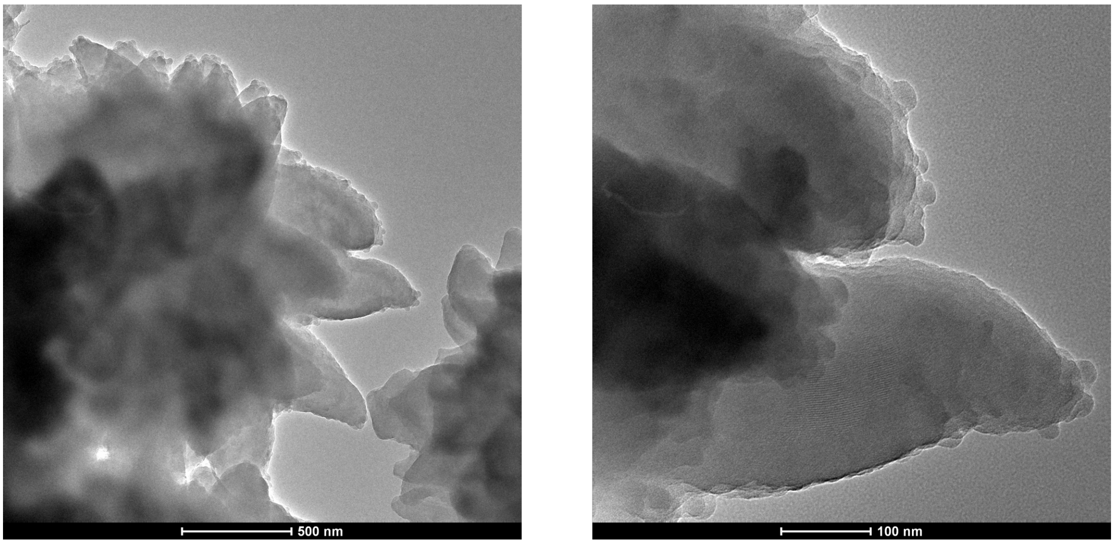


**Fig. 14** TEM images of 30%PEG@BT-COF (left and right). The right is the magnified TEM image, proving the invisibility of crystalline domains when the mesopores of the BT-COF were filled up with PEG.

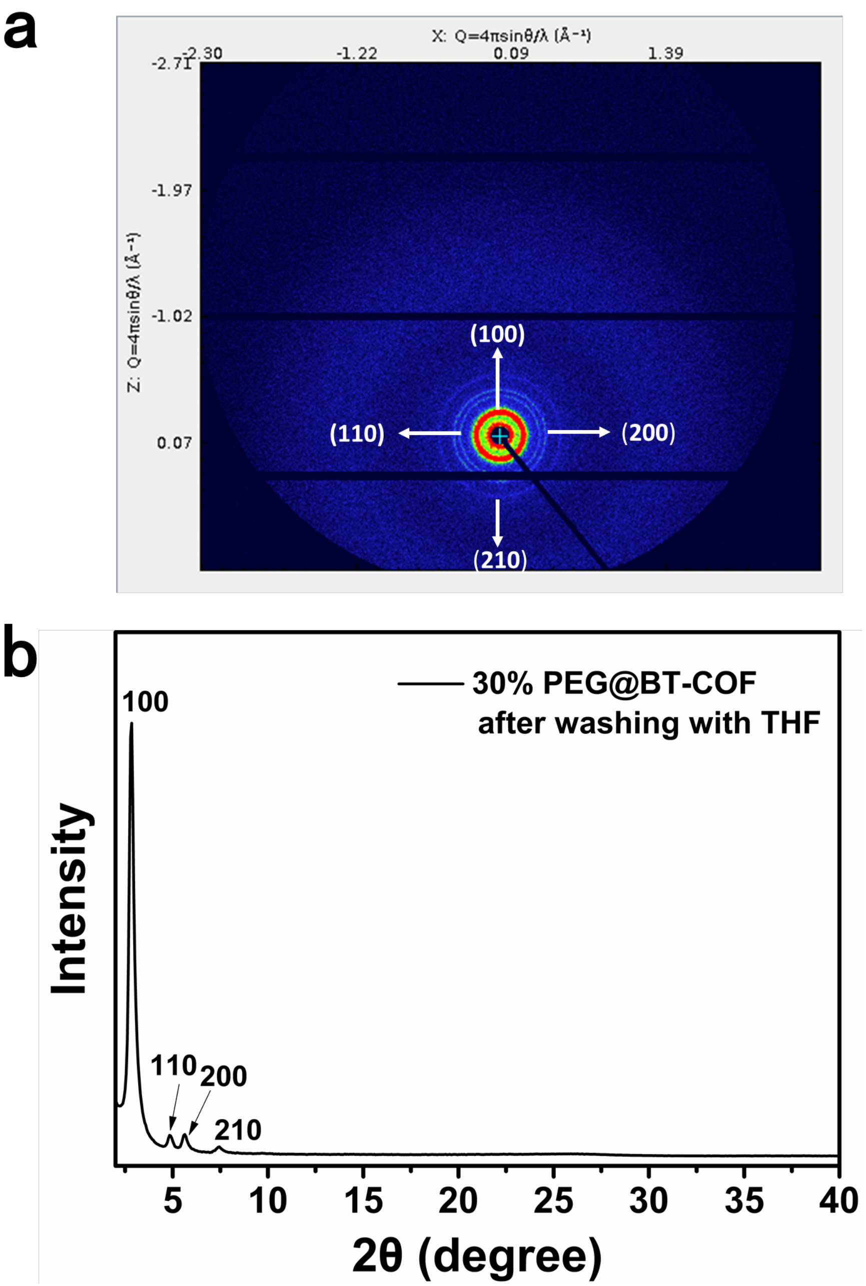


**Fig. 15** WAXS 2D pattern (a) and profile (b) of 30%PEG@BT-COF that was thoroughly extracted with THF for 3 days by Soxhlet. All of the peaks could be assigned to the (100), (110), (200) and (210) lattice planes of BT-COF.

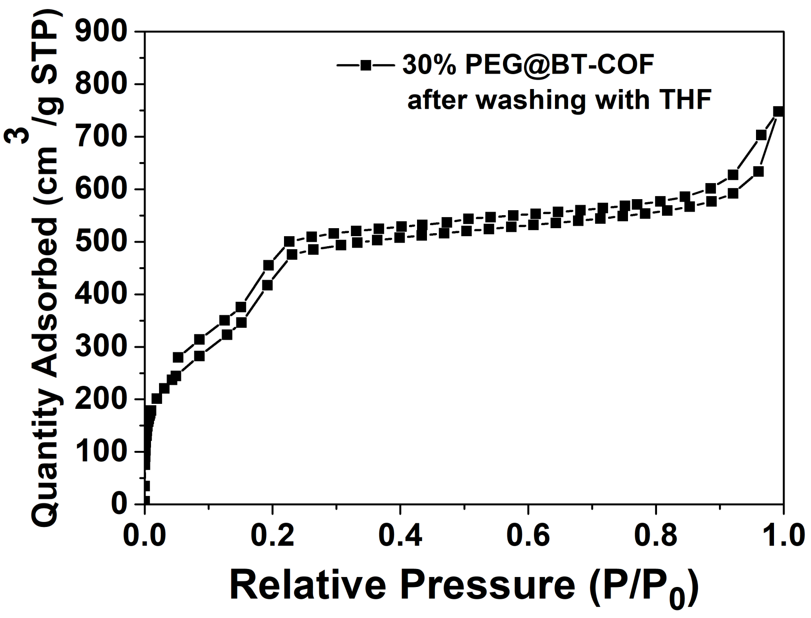


**Fig. 16** $N_2$ sorption isotherm of 30%PEG@BT-COF that was thoroughly extracted with THF for 3 days by Soxhlet. The BET surface area is recovered to be 1286 $m^2$ $g^{-1}$ as the loaded PEG has been successfully extracted from 1D pore channels of BT-COF.

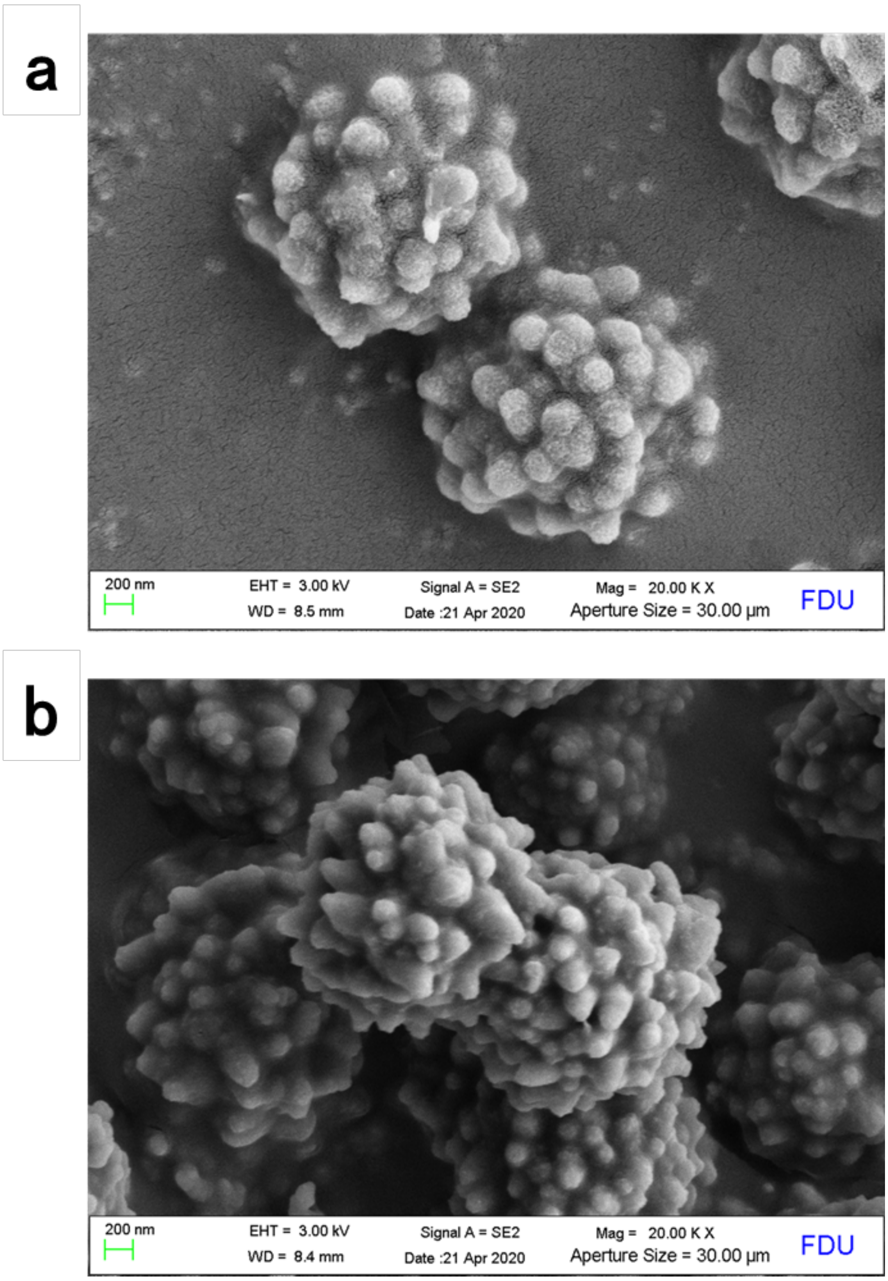


**Fig. 17** SEM images of 30%PEG@BT-COF before (a) and after (b) sonication in ethanol for 30 min.

**Table 2.** FWHM ($R^2$) and average crystallite domain sizes computed by Scherrer equation.

| | FWHM ($R^2$)[a] | Average crystallite domain sizes (nm) |
|---|---|---|
| BT-COF | 0.1981 (0.9910) | 44.6 |
| Recycled BT-COF | 04090 (0.9977) | 21.6 |
| 30%PEG@BT-COF (PEG = 20k) | 0.2299 (0.9890) | 38.4 |
| Post-extracted PEG@BT-COF[b] | 0.2784 (0.9778) | 31.8[b] |
| Re-30%PEG@BT-COF | 0.2670 (0.9935) | 33.1 |
| Post-extracted re-30%PEG@BT-COF[b] | 0.2997 (0.9810) | 29.6[b] |

[a]FWHM($R^2$) values were obtained from Supplementary Fig. 18.

[b]The samples were extracted by THF to remove the loaded PEG chains. As compared with those before solvent post-extraction, the average crystallite sizes decrease a little. This is possibly resulted from the solvation exfoliation effect.

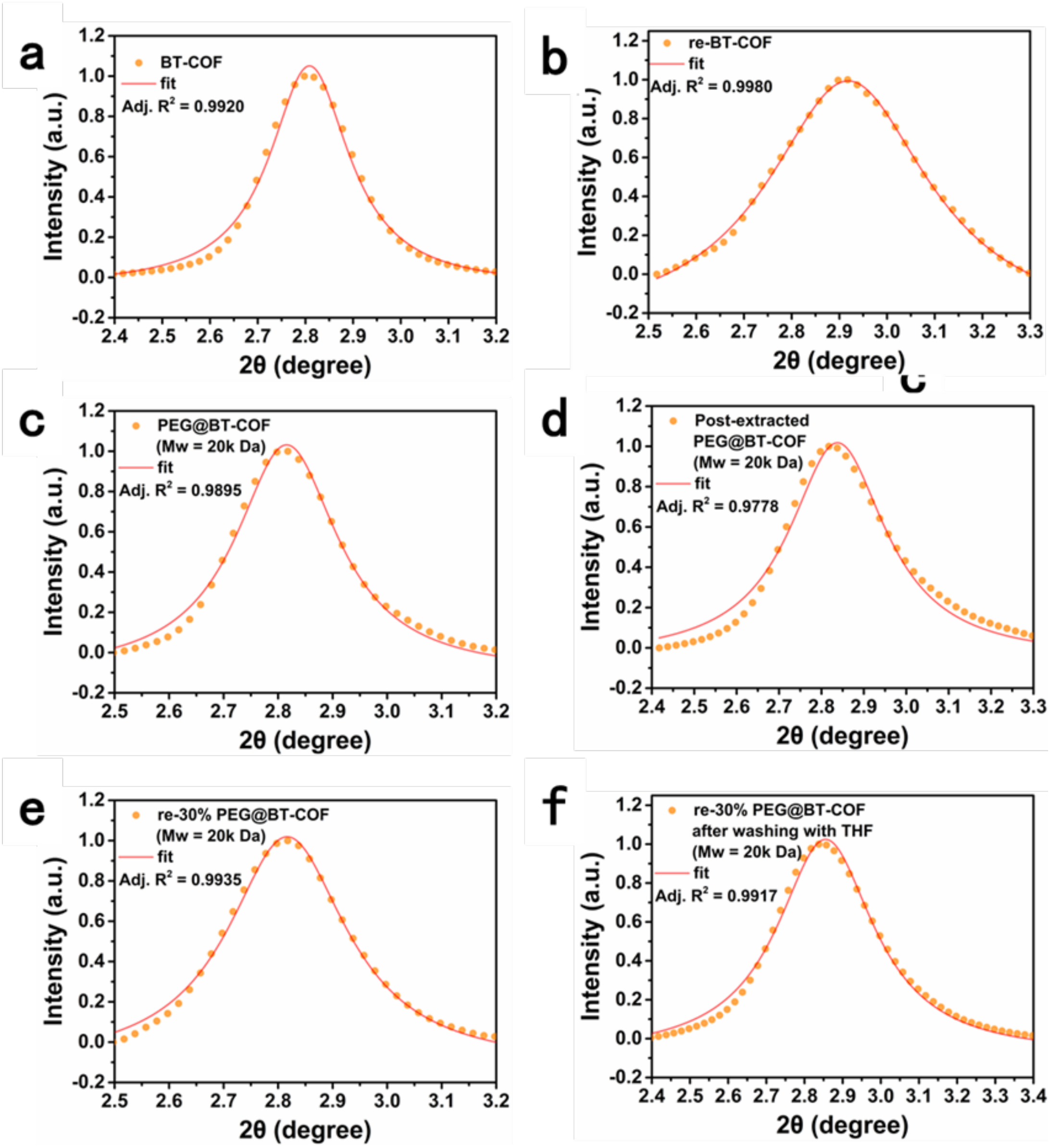


**Fig. 18** The (100) X-ray scattering peaks fitted by Lorenza function for obtaining the full width at half maximum (FWHM).

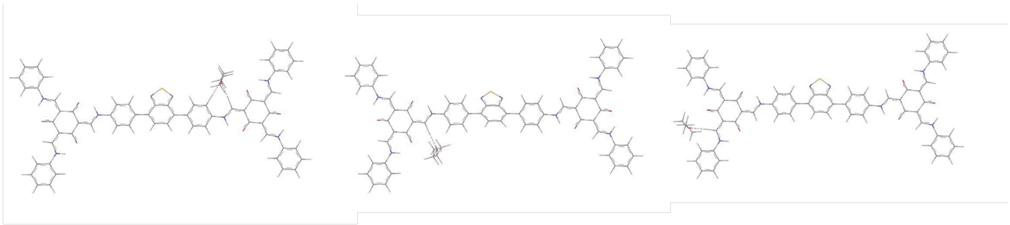

0.47 0.46 0.46

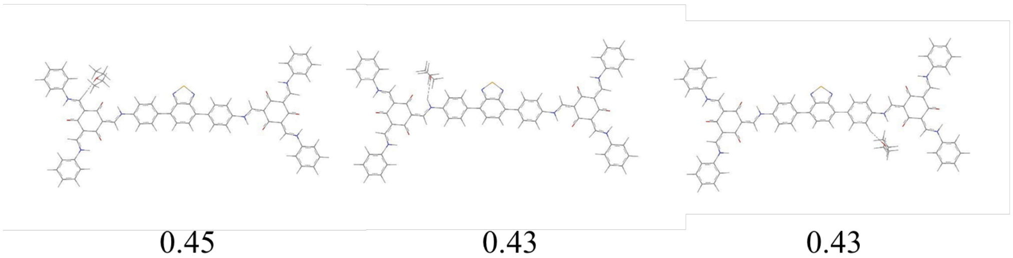

0.45 0.43 0.43

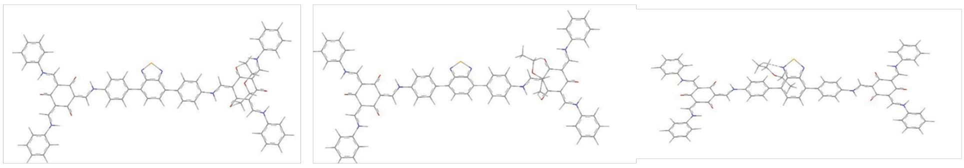

0.50 0.48 0.41

**Fig. 19** Schematic binding positions of PEG cutout model-1, P1, to the BT(G1), as well as the binding energy, in eV, calculated using CAM-B3LYP-D3BJ/def2SVP. The top two rows correspond to the binding to the sideways, while the bottom row corresponds to the binding on top of the COF.

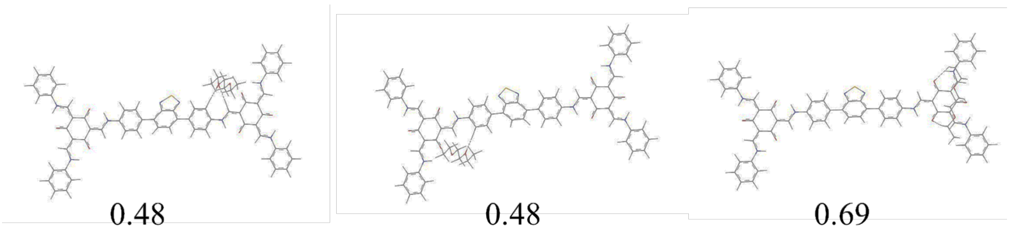


**Fig. 20** Schematic binding positions of PEG cutout model-2, P2, to the BT(G1), as well as the binding energy, in eV, calculated using CAM-B3LYP-D3BJ/def2SVP. The left and middle columns correspond to the binding on the side, while the right column corresponds to the binding on top of the COF.

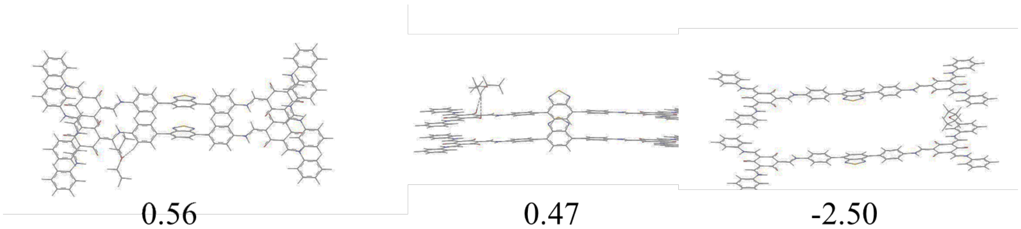

0.56 0.47 -2.50

**Fig. 21** Schematic binding positions of P1 to the BT(D1), as well as the binding energy, in eV, calculated using CAM-B3LYP-D3BJ/def2SVP. The left column corresponds to binding on the side, the central column corresponds to the binding on top of the COF, and the right column corresponds to PEG entering in between the π-stacked dimer.

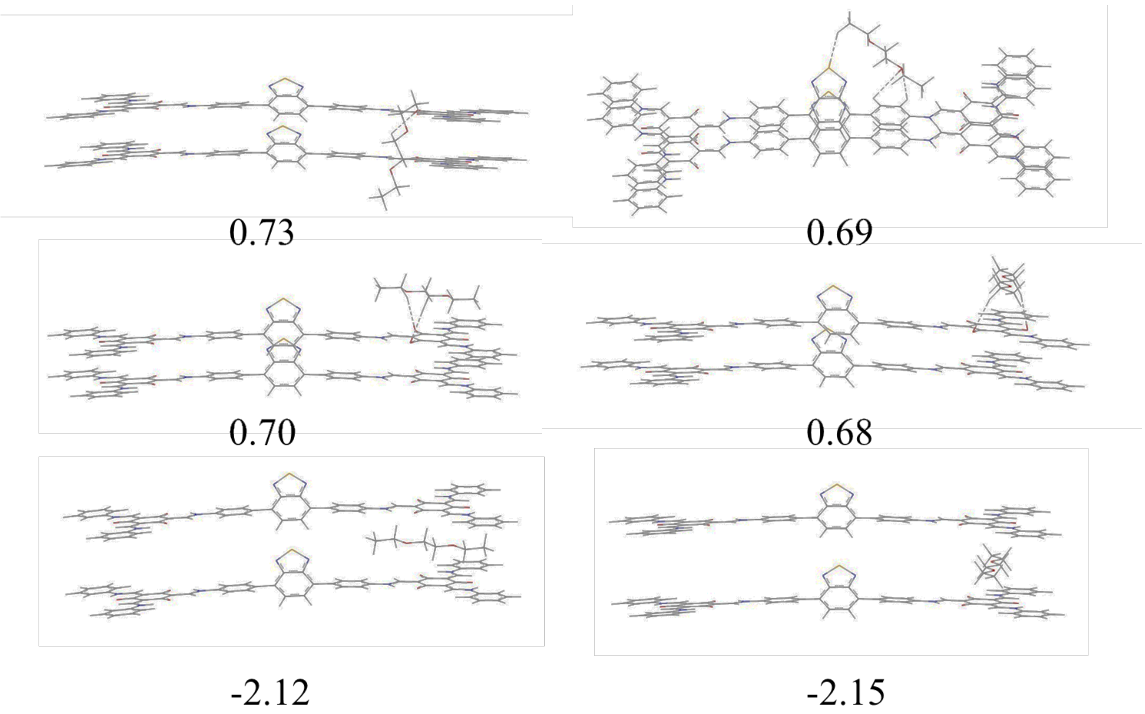


**Fig. 22** Schematic binding positions of P2 to the BT(D1), as well as the binding energy, in eV, calculated using CAM-B3LYP-D3BJ/def2SVP. In the first row, we present the stable geometries for the sideways binding, while on the second row, we present the results for on the surface binding, while on the bottom row, we present the results for placing P2 in between the π-stacked dimer.

**Table 3.** Dimer binding energy calculated using CAM-B3LYP-D3BJ/def2SVP.

| Entry | System | Energy (eV) |
|---|---|---|
| 1 | 2 Bare BT(G1) | 3.48 |
| 2 | Bare BT(G1)+BT(G1)•••P1 sideways | 3.57 |
| 3 | Bare BT(G1)+BT(G1)•••P1 on top | 3.44 |
| 4 | Bare BT(G1)+BT(G1)•••P2 sideways | 3.73 |
| 5 | Bare BT(G1)+BT(G1)•••P2 on top | 3.49 |

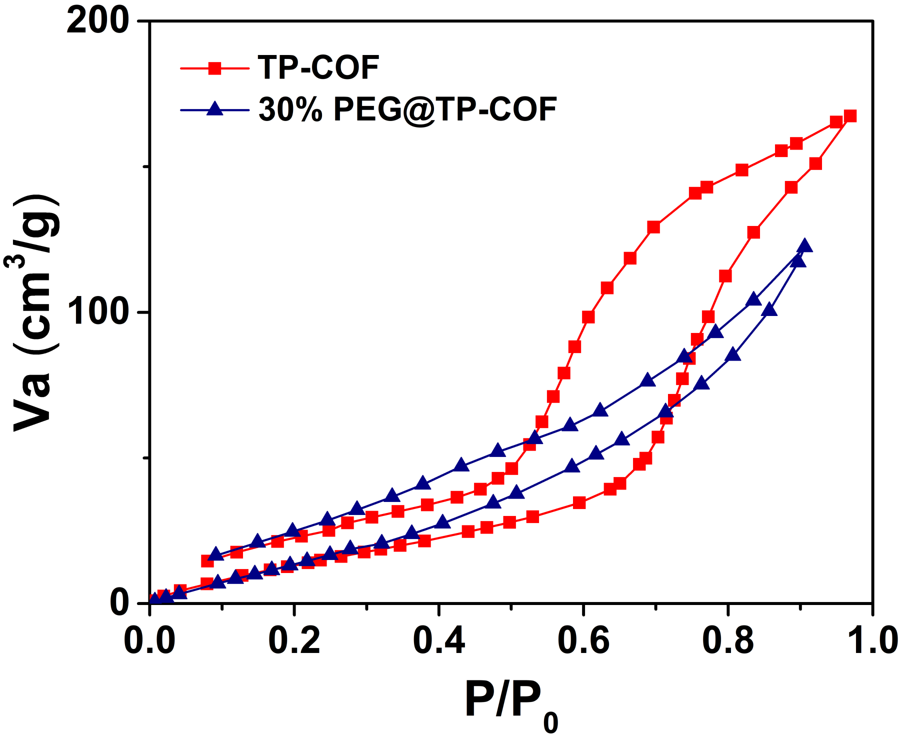


**Fig. 23** Water uptake profiles of TP-COF and 30%PEG@TP-COF at 298K, respectively.

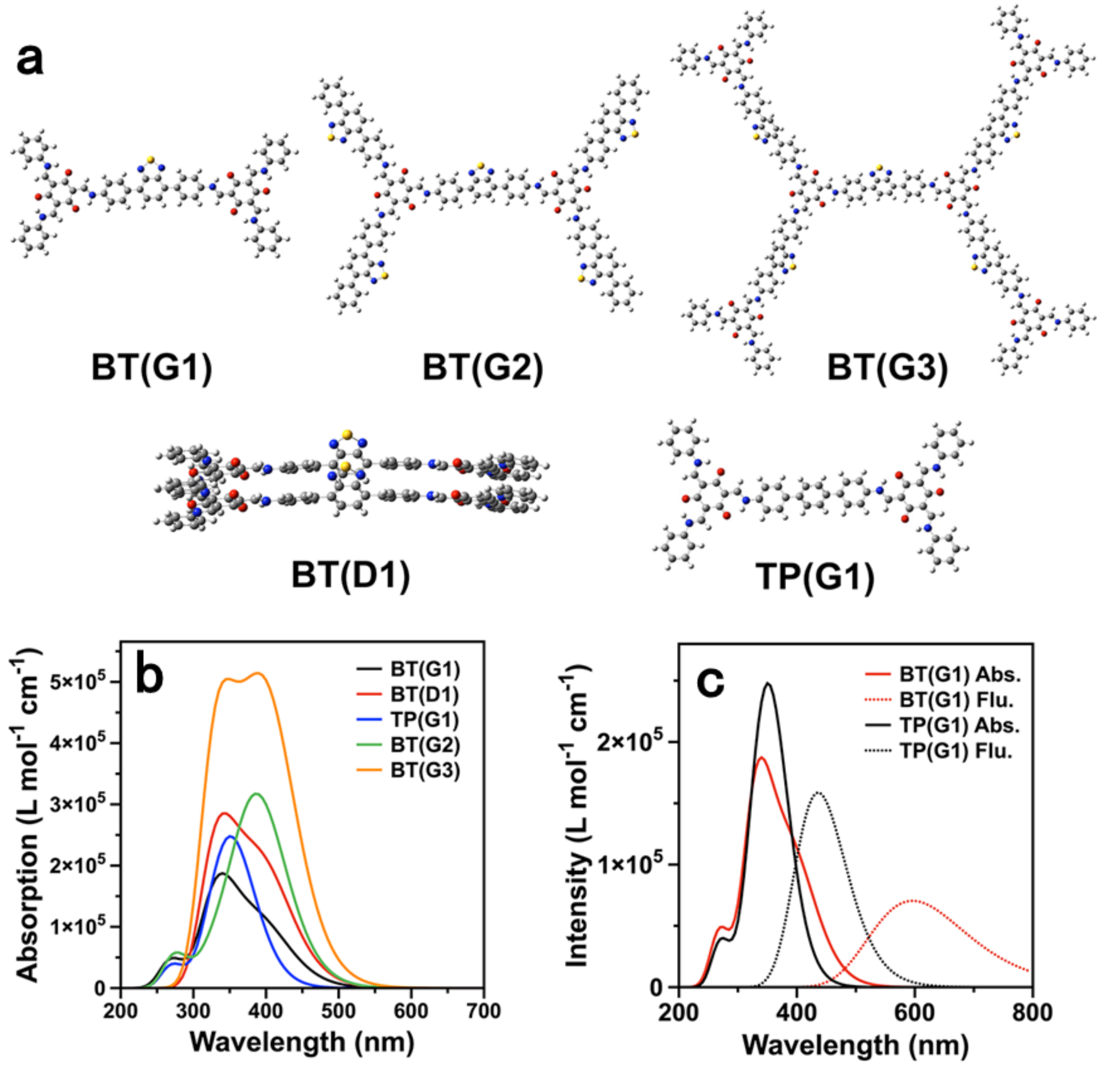


**Fig. 24** (a) Schematic diagram of the cutout models used for BT-COF and TP-COF. The simulated spectra: (b) absorption from the ground electronic state for the different cutout models of BT-COF and TP-COF, and (c) absorption and fluorescence emission for the smallest cutout model of BT-COF and TP-COF. All the spectra were modeled using the peak position and intensity obtained from TD-CAMB3LYP/def2SVP. Gaussian function with an artificial broadening of 0.2 eV was used to model the spectra.

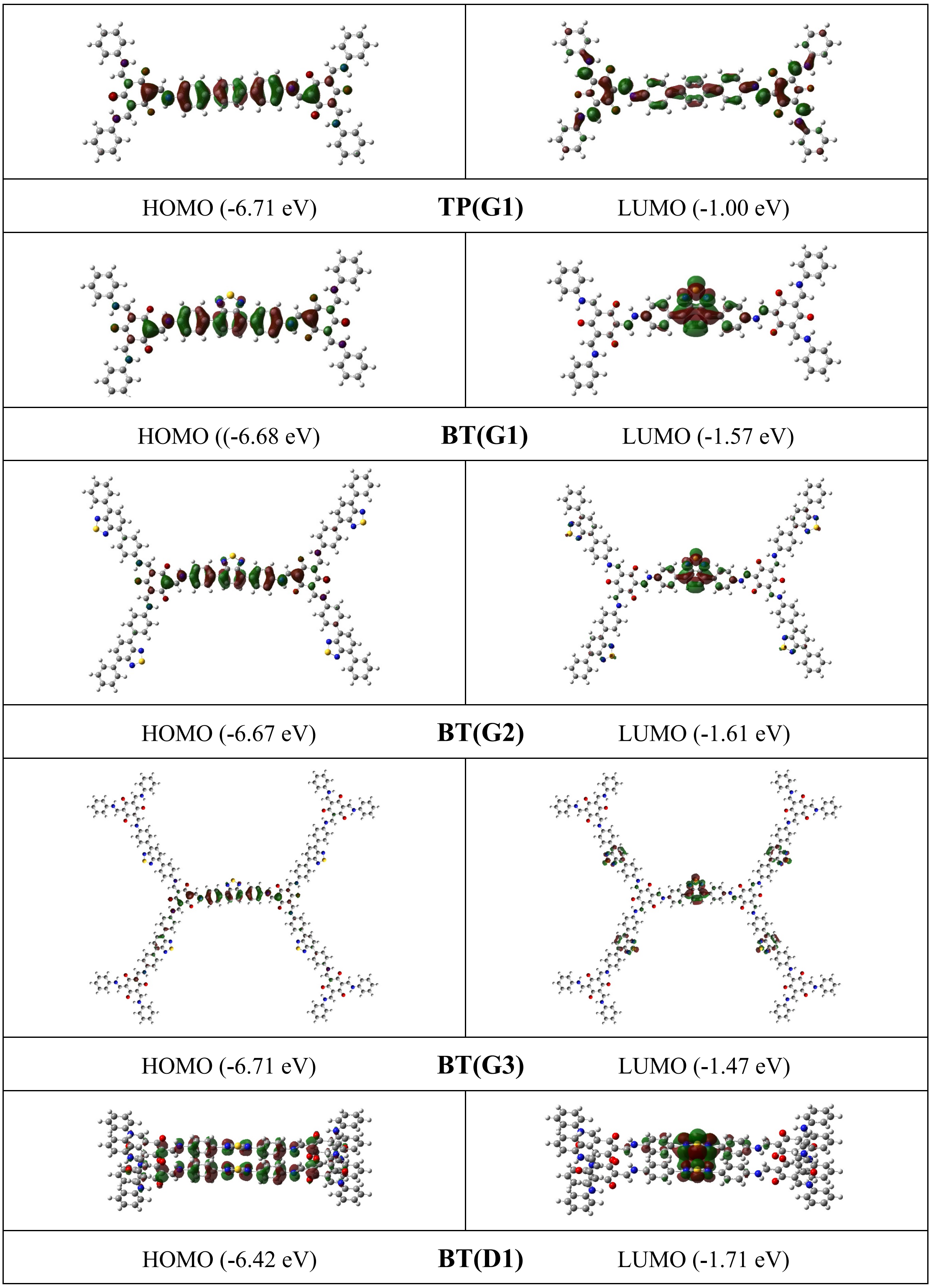


**Fig. 25** DFT calculation for the HOMO and LUMO orbitals and energy levels of the cutout models including G1, G2, G3 and D1 for the BT-COF and G1 for the TP-COF, respectively.

**Table 4.** Calculated peak position, in nm, and oscillator strength from the ground electronic state minima to the first absorbing excited state, and the fluorescence emission peak position and oscillator strength from the first excited state minima to the ground electronic state. All calculations were performed using TD-CAMB3LYP/def2SVP.

| | Absorbance (nm) | Oscillator Strength |
|---|---|---|
| BT(G3) SMD | 404 | 4.41 |
| BT(G2) SMD | 400 | 3.21 |
| BT(G1) SMD | 397 | 2.36 |
| BT(D1) SMD | 399 | 4.46 |
| TP(G1) SMD | 361 | 4.04 |
| | Fluorescence emission (nm) | Oscillator Strength |
| BT(G1) SMD | 596 | 1.74 |
| TP(G1) SMD | 436 | 3.92 |

**Table 5.** The calculated potential with respect to standard hydrogen electrode at pH=0 for the oxidation reactions of ascorbic acid and water calculated using CAMB3LYP-D/def2SVP SMD. The geometries of the hydrated clusters are given in Supplementary Fig. 50.

| | Potential (V) |
|---|---|
| $HA \ldots . H_2O + H^+ + e^- \rightarrow H_2A \ldots . H_4O_2$ | 0.88 |
| $DHAD \ldots . H_2O + H^+ + e^- \rightarrow HA \ldots . H_4O_2$ | -0.51 |
| $DHAD \ldots . H_2O + 2H^+ + 2e^- \rightarrow H_2A \ldots . H_4O_2$ | 0.37 |
| $\frac{1}{4}O_2 + H^+ + e^- \rightarrow \frac{1}{2}H_2O$ | 1.17 |

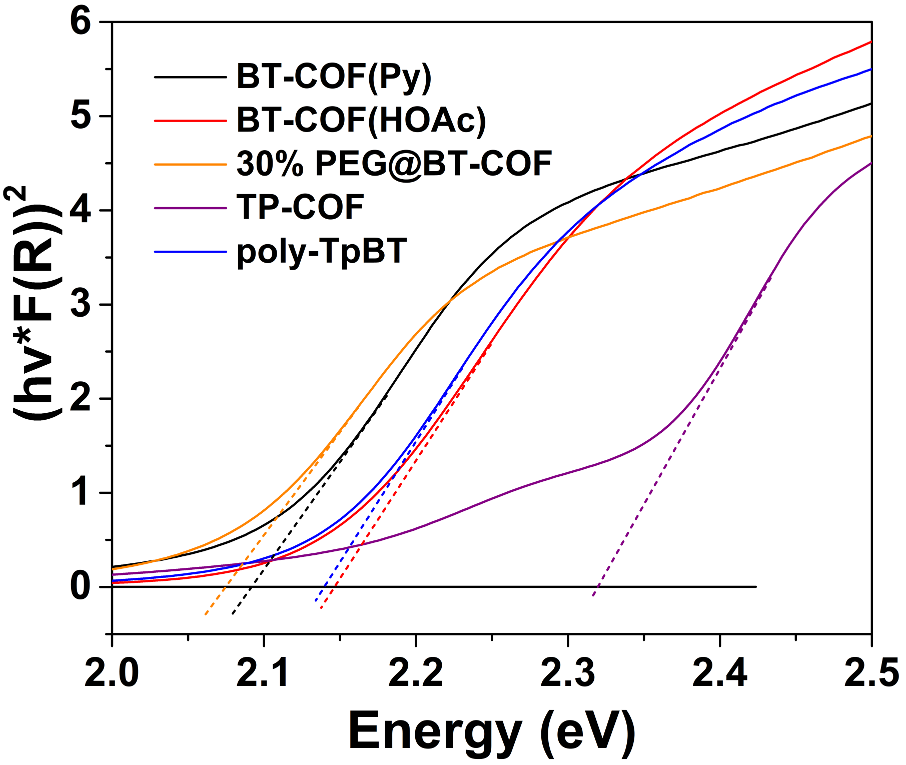


**Fig. 26** Tauc plots of BT-COF, BT-COF(HOAc), Poly(TpBT), TP-COF and 30%PEG@BT-COF, respectively.

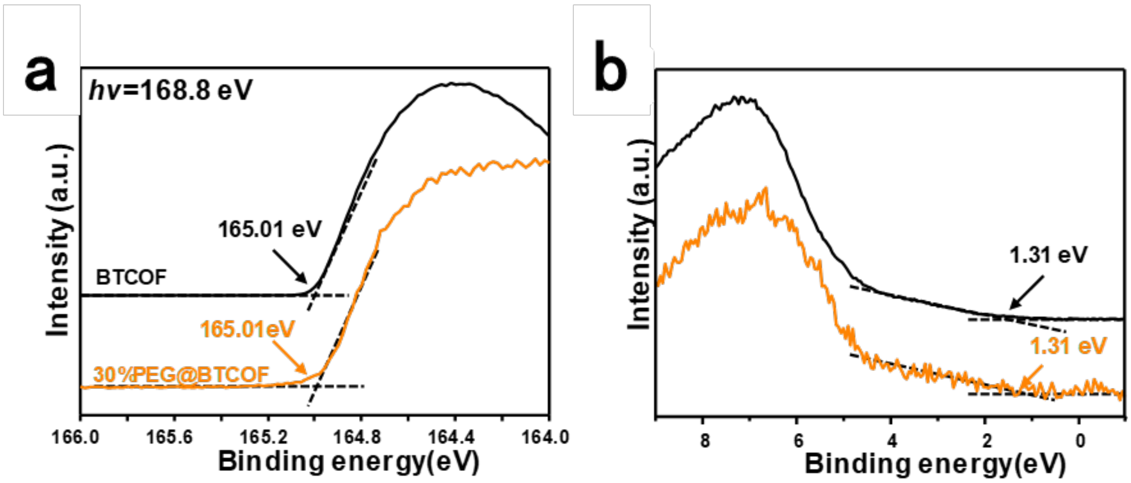


**Fig. 27** Secondary electron cutoff (a) and valence band spectra (b) of the BT-COF and 30%PEG@BT-COF measured by synchrotron radiation photoemission spectroscopy (SRPES).

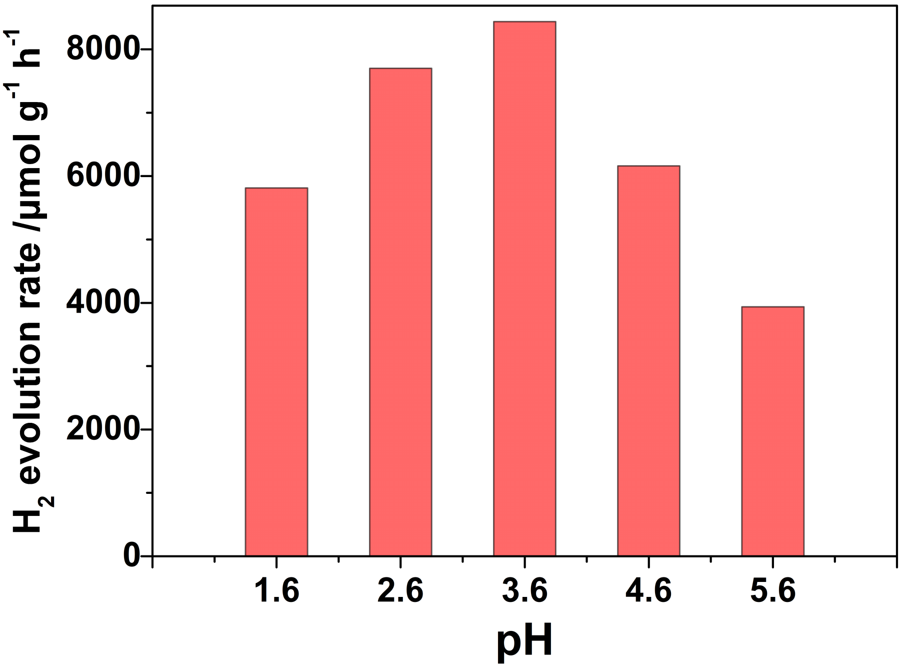


**Fig. 28** Photocatalytic $H_2$ evolution rates for the BT-COF at different pH values under λ > 420 nm irradiation using ascorbic acid as a sacrificial electron donor in the presence of Pt nanoparticles (3.5wt%). The pH value of 0.1 M ascorbic acid solution is 2.6.

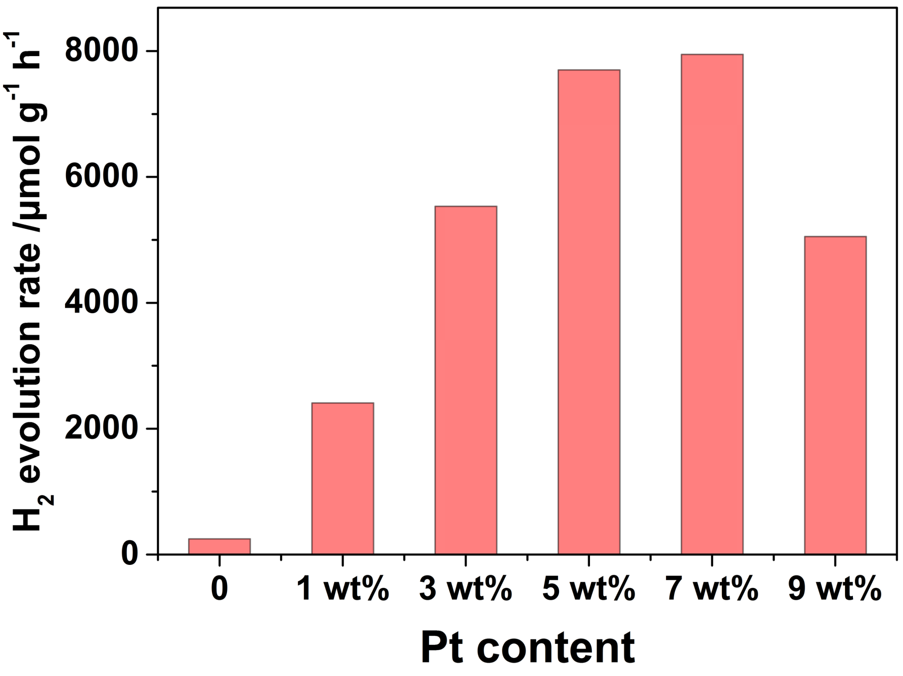


**Fig. 29** Photocatalytic $H_2$ evolution rates of the BT-COFs, on which the different feeding amounts of Pt cocatalysts were deposited.

**Table 6.** Pt contents of the different samples measured by ICP-AES.

| | Feeding content (wt%) | ICP content (wt%) |
|---|---|---|
| BT-COF | 3 | 1.87 |
| BT-COF | 5 | 3.54 |
| BT-COF | 7 | 5.10 |
| 30%PEG@BT-COF | 5 | 3.73 |

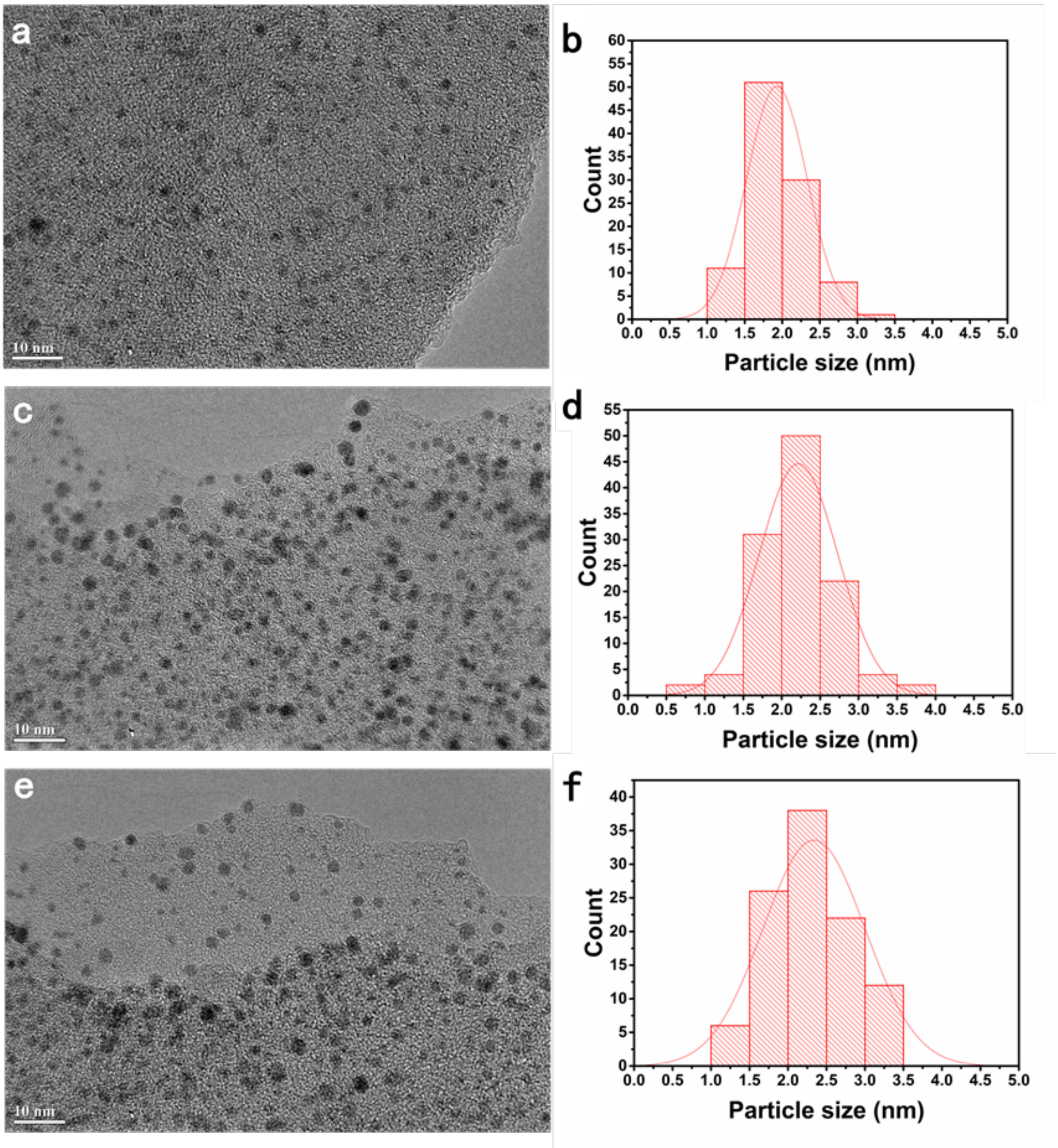


**Fig. 30** TEM images and statistical size distributions of the Pt nanoparticles photo-deposited on the BT-COF (a,b), 30%PEG-2000@BT-COF (c,d) and 30%PEG-20k@BT-COF (e,f), respectively.

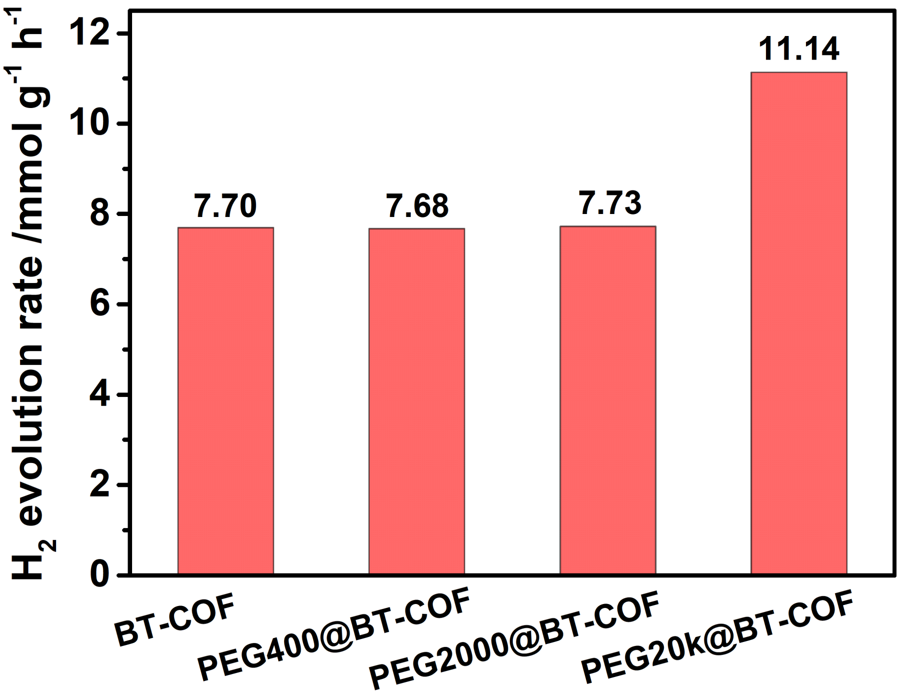


**Fig. 31** Photocatalytic $H_2$ evolution rates of the BT-COF and three kinds of the PEG@BT-COFs, respectively, under λ > 420 nm irradiation for 8 h, using ascorbic acid as a sacrificial electron donor (0.1 M) in the presence of Pt nanoparticle (~3.7wt%). The feeding amounts of PEG400, PEG2000 and PEG20k are all 30wt% relative to the BT-COF.

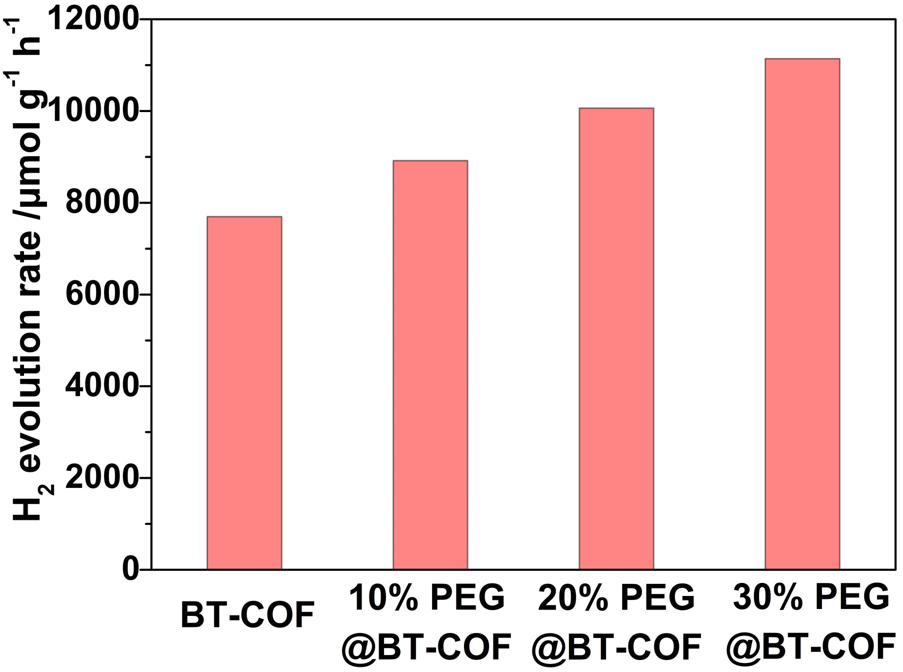


**Fig. 32** Photocatalytic $H_2$ evolution rates of the BT-COF and 10%-, 20%- and 30%PEG-20k@BT-COF, respectively, under $\lambda > 420$ nm irradiation for 8 h, using ascorbic acid as a sacrificial electron donor (0.1 M) in the presence of Pt nanoparticle (3.7wt%).

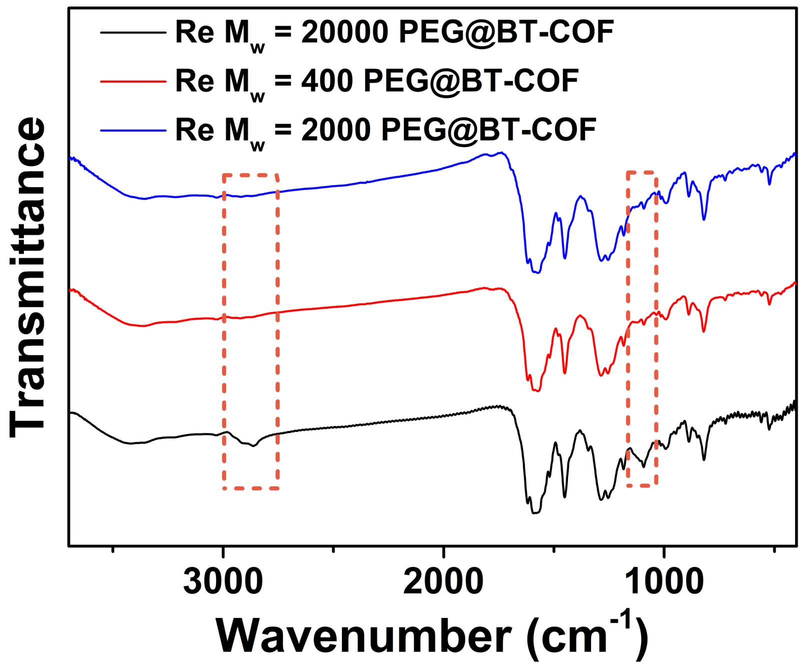


**Fig. 33** FT IR spectra of the recycled PEG20k@BT-COF, PEG400@BT-COF and PEG2000@BT-COF after 48-h photocatalysis cycles, respectively.

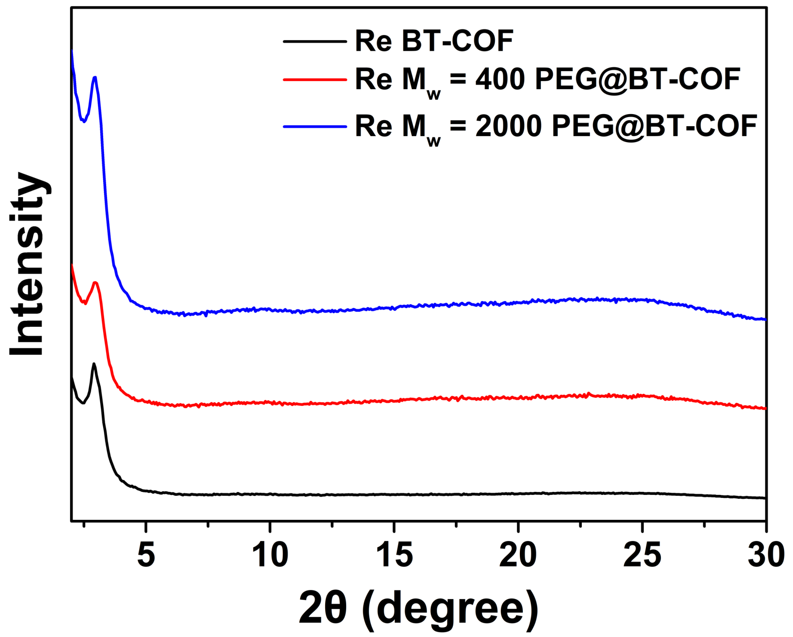


**Fig. 34** WAXS profiles of the recycled BT-COF, PEG400@BT-COF and PEG2000@BT-COF after 48-h photocatalysis cycles, respectively.

**Table 7.** Photocatalytic hydrogen evolution performances of the different COF-based photocatalysts.

| Photocatalyst | Co-catalyst | SED | Illumination | HER (mmol $g^{-1}$ $h^{-1}$) | AQE | Ref. |
|---|---|---|---|---|---|---|
| TFPT-COF | 3% Pt | 1 wt% sodium ascorbate | > 420nm | 0.23 | | [28] |
| TFPT-COF | 3% Pt | 10 vol% TEOA | > 420nm | 1.97 | 2.2-3.9% at 500nm | [28] |
| $N_0$-COF | 3% Pt | 1 vol% TEOA pH = 7 | > 420nm | 0.023 | 0.001% at 450nm | [29] |
| $N_1$-COF | 3% Pt | 1 vol% TEOA pH = 7 | > 420nm | 0.090 | 0.077% at 450nm | [29] |
| $N_2$-COF | 3% Pt | 1 vol% TEOA pH = 7 | > 420nm | 0.438 | 0.19% at 450nm | [29] |
| $N_3$-COF | 3% Pt | 1 vol% TEOA pH = 7 | > 420nm | 1.703 | 0.44% at 450nm | [29] |
| $N_1$-COF | Co-1 | 1 vol% TEOA pH = 8 | AM1.5 | 0.100 | | [30] |
| $N_2$-COF | Co-1 | 1 vol% TEOA pH = 8 | AM1.5 | 0.782 | 0.16% at 400nm | [30] |
| $N_2$-COF | Co-2 | 1 vol% TEOA pH = 10 | AM1.5 | 0.414 | | [30] |
| $N_3$-COF | Co-1 | 1 vol% TEOA pH = 8 | AM1.5 | 0.163 | | [30] |
| COF-42 | Co-1 | 1 vol% TEOA pH = 8 | AM1.5 | 0.233 | | [30] |
| PTP-COF | 3% Pt | 1 vol% TEOA pH = 7 | AM1.5 | 0.084 | | [31] |
| TP- EDDA-COF | 3% Pt | 10 vol% TEOA | > 395 nm | 0.03 | | [32] |
| TP- BDDA-COF | 3% Pt | 10 vol% TEOA | > 395 nm | 0.33 | 1.3% at 420 nm; 1.8% at 520nm | [32] |
| FS-COF | 8% Pt | 0.1 M ascorbic acid | > 420 nm | 10.1 | 3.2% at 420 nm | [21] |
| S-COF | 8% Pt | 0.1 M ascorbic acid | > 420 nm | 4.44 | | [21] |
| TP-COF | 8% Pt | 0.1 M ascorbic acid | > 420 nm | 1.60 | | [21] |
| A-TEBPY-COF | 3% Pt | 10 vol% TEOA pH = 7 | AM1.5 | 0.098 | | [33] |
| A-TENPY-COF | 3% Pt | 10 vol% TEOA pH = 7 | AM1.5 | 0.022 | | [33] |
| A-TEPPY-COF | 3% Pt | 10 vol% TEOA pH = 7 | AM1.5 | 0.006 | | [33] |

| | | | | | | |
|---|---|---|---|---|---|---|
| CTF-HUST-C1 | 3% Pt | 10 vol% TEOA | > 420 nm | 5.1 | | [34] |
| CTF-HUST-C5 | 3% Pt | 10 vol% TEOA | > 420 nm | 2.4 | | [34] |
| CTF-HUST-C6 | 3% Pt | 10 vol% TEOA | > 420 nm | 0.65 | | [34] |
| $SP^2$-COF | 3% Pt | 10 vol% TEOA | > 420 nm | 1.36 | | [35] |
| $SP^2$-$COF_{ERDN}$ | 3% Pt | 10 vol% TEOA | > 420 nm | 2.12 | 0.47% at 520 nm | [35] |
| g-$C_{40}N_3$-COF | 3% Pt | 10 vol% TEOA | > 420 nm | 4.12 | 4.84% at 420 nm | [36] |
| TP-COF | 6% PVP-Pt | 0.054 M ascorbic acid | > 420 nm | 8.42 | 0.4% at 420 nm | [37] |
| CTF-HUST-A1-$^t$BuOK | 3% Pt | 10 vol% TEOA | > 420 nm | 9.2 | 7.4% at 420 nm | [38] |
| **BT-COF** | **3.5% Pt** | **0.1 M ascorbic acid** | **> 420 nm** | **7.70** | **7.53% at 420 nm** | **This work** |
| **30%PEG@BT-COF** | **3.7% Pt** | **0.1 M ascorbic acid** | **> 420 nm** | **11.14** | **11.20% at 420 nm** | **This work** |

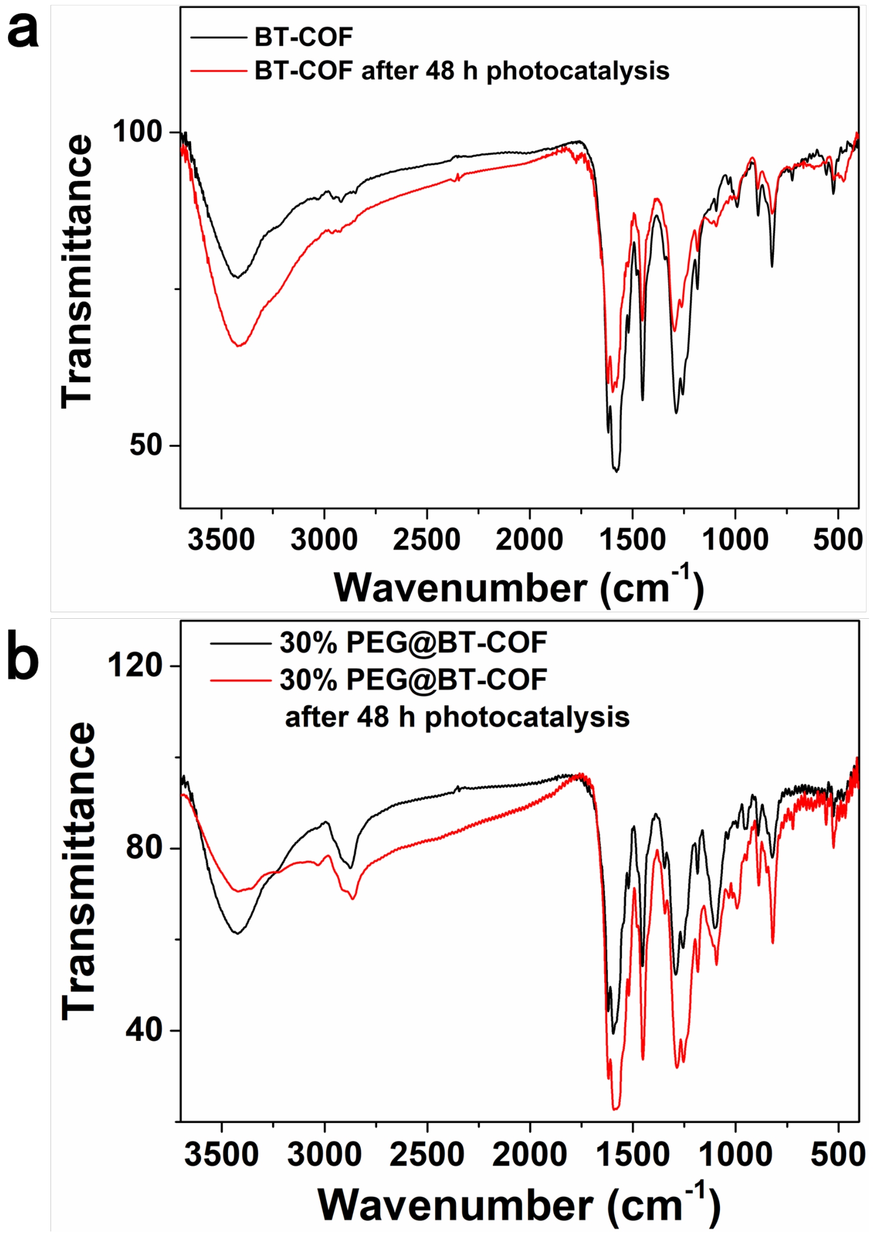


**Fig. 35** FT IR spectra of BT-COF (a) and 30%PEG@BT-COF (b) before (black) and after (red) the 48-h photocatalytic test.

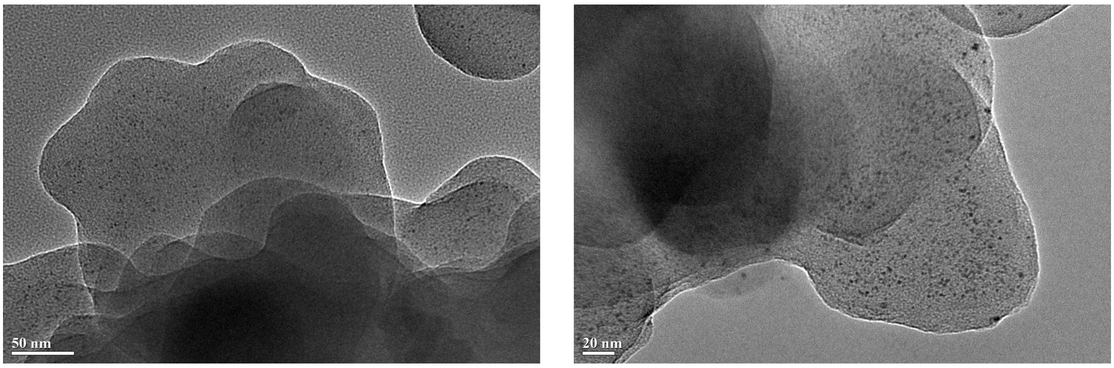


**Fig. 36** TEM images of the 30%PEG@BT-COF recycled after the 48-h hydrogen evolution test under visible light (λ > 420 nm). The right image shows the magnified view of the representative 30%PEG@BT-COF in the presence of Pt nanoparticles.

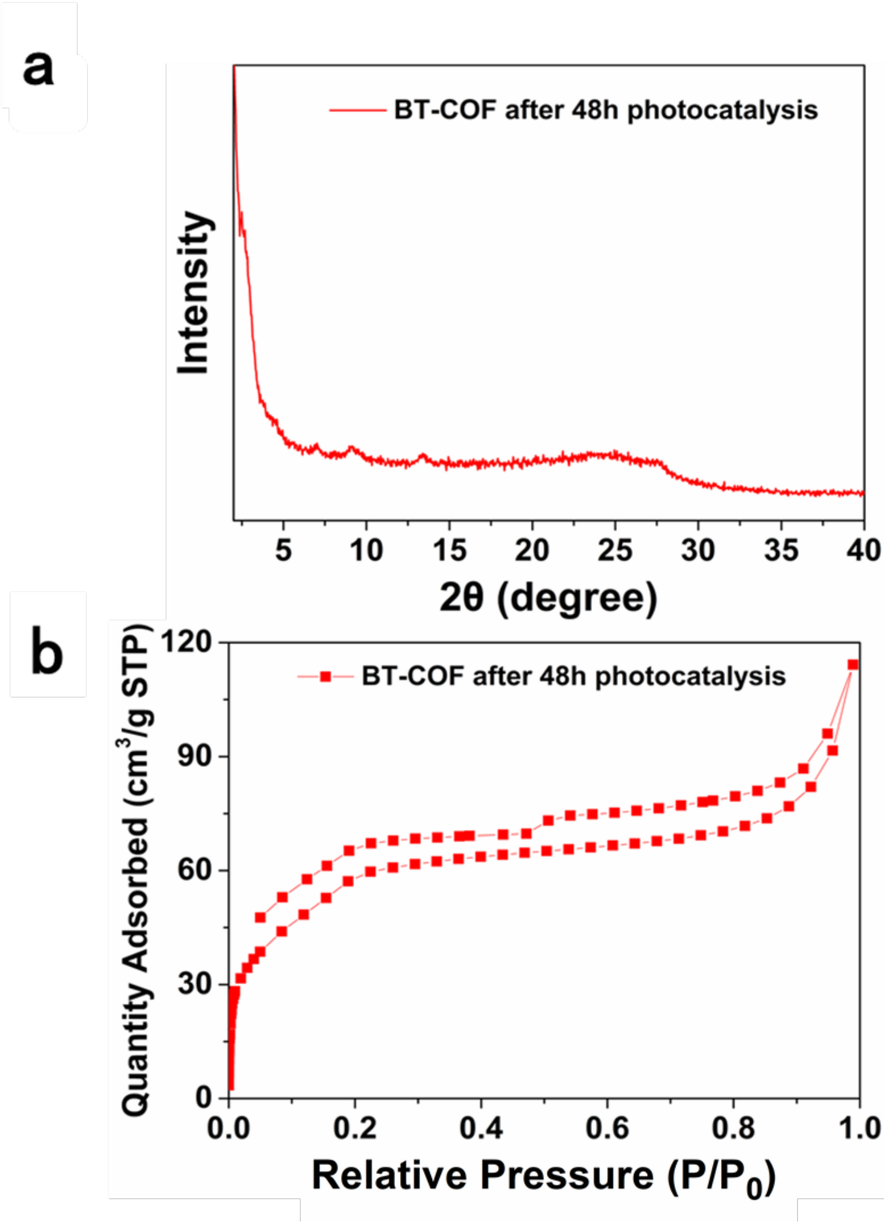


**Fig. 37** PXRD pattern (a) and $N_2$ sorption isotherms (b) of the BT-COF recycled after the 48-h photocatalytic test.

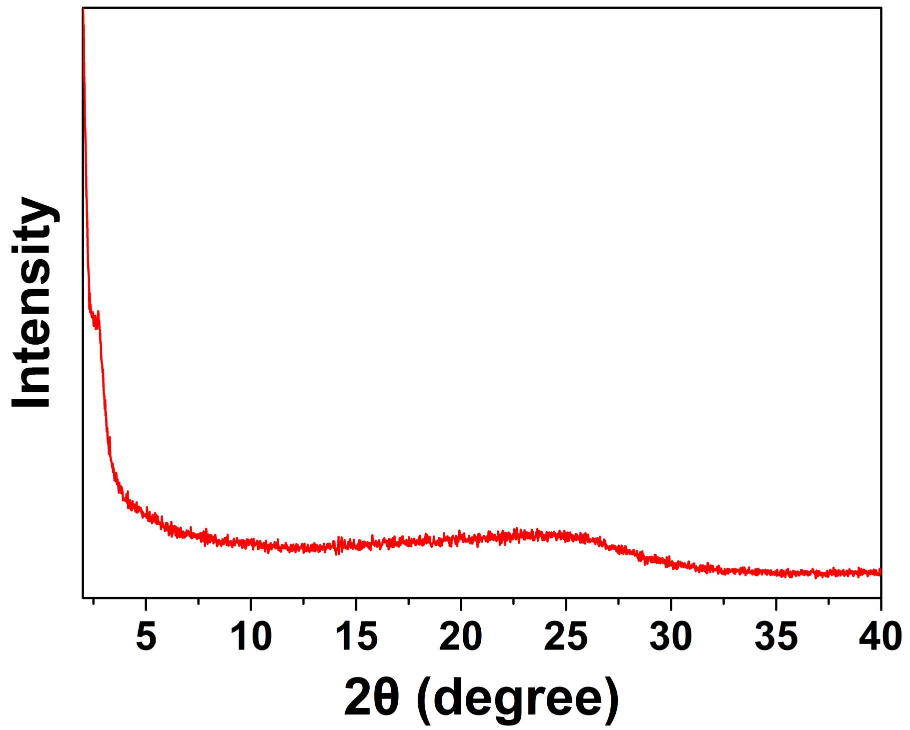


**Fig. 38** PXRD patterns of the recycled 30%PEG@BT-COF obtained after the 48-h photocatalytic test.

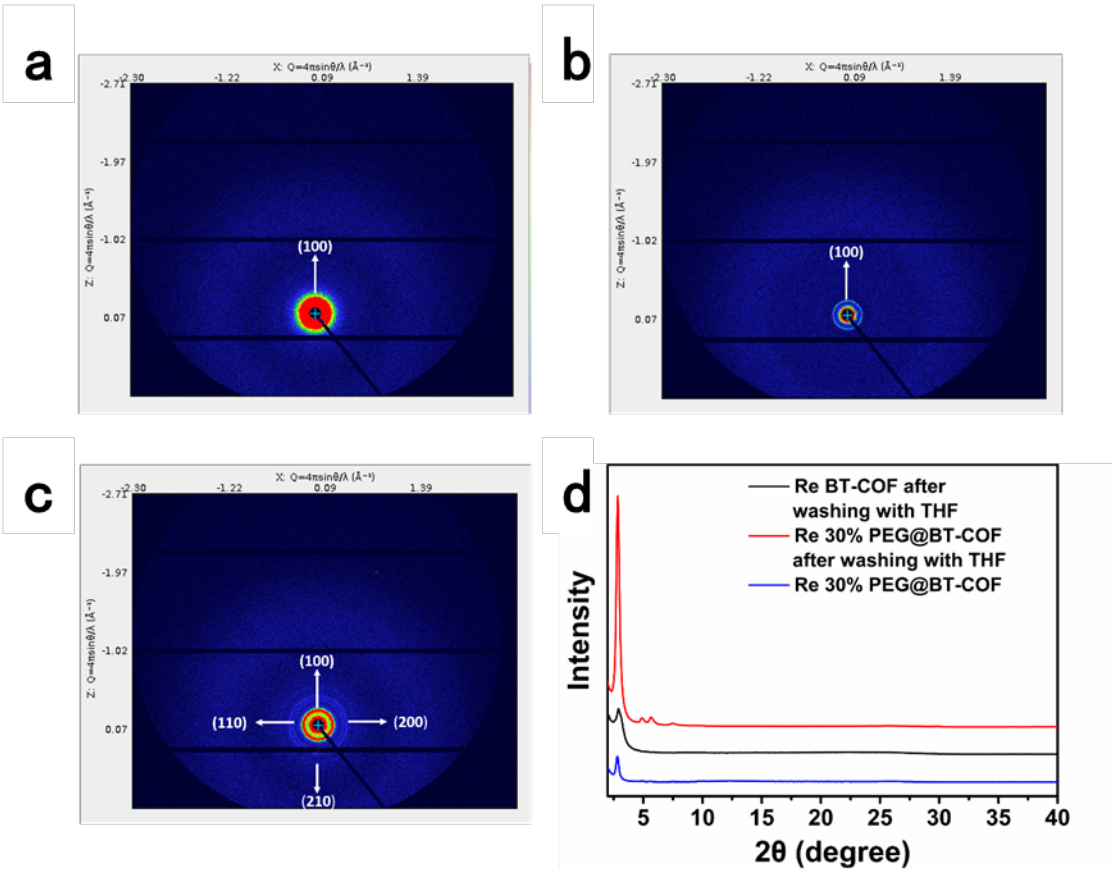


**Fig. 39** WAXS 2D pattern of 48-h recycled BT-COF after Soxhlet extraction with THF for 3 days (a), WAXS 2D pattern of 48-h recycled 30%PEG@BT-COF before (b) and after (c) Soxhlet extraction with THF for 3 days, WAXS profile (d) of the corresponding recycled photocatalysts.

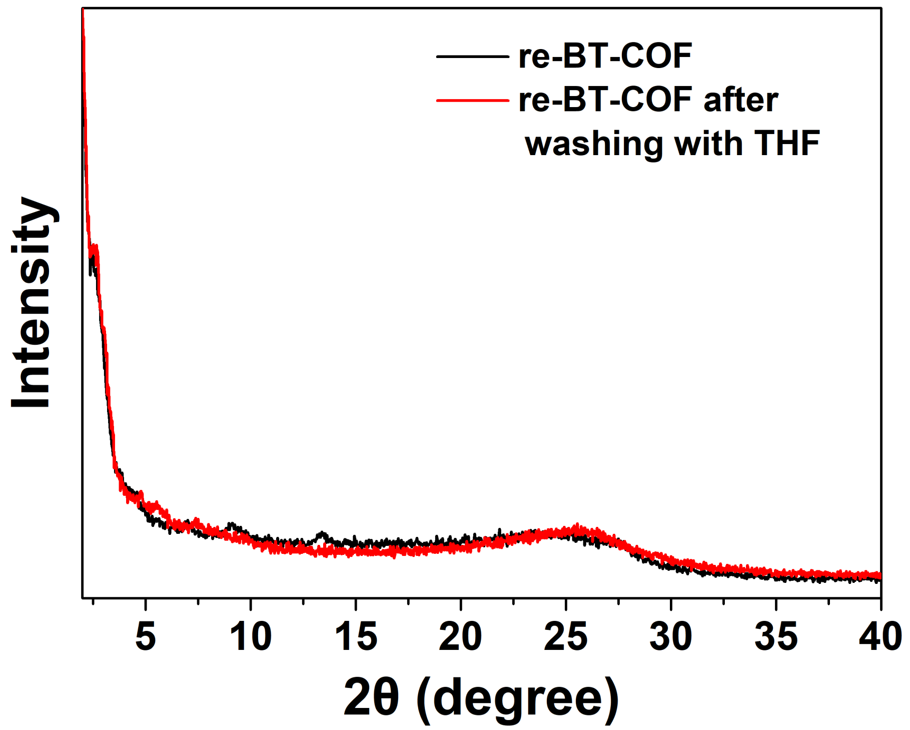


**Fig. 40** PXRD patterns of the 48-h recycled BT-COF before and after Soxhlet extraction with THF for 3 days.

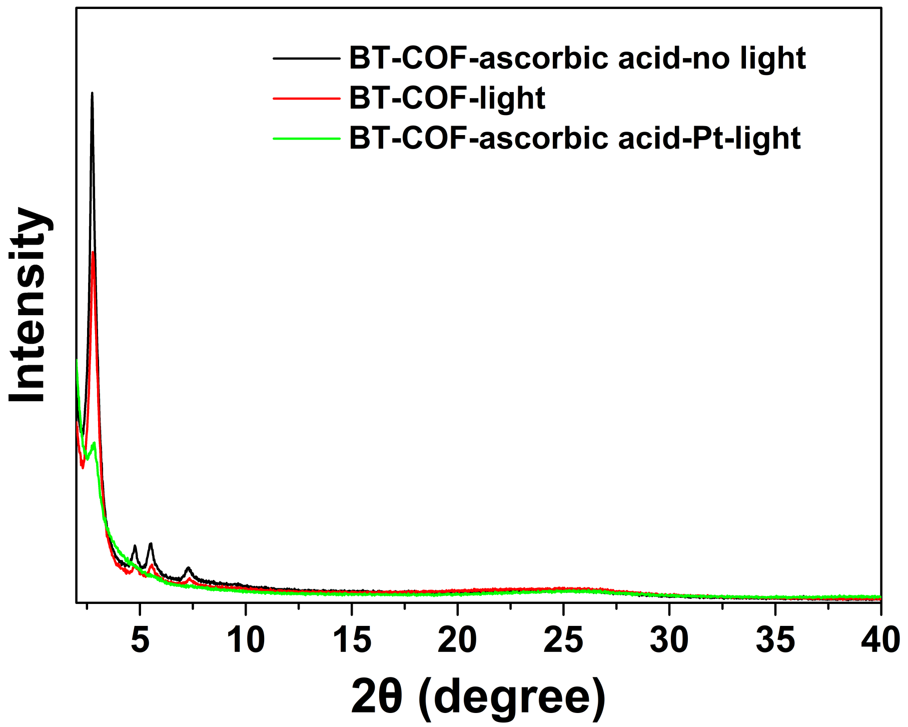


**Fig. 41** PXRD patterns of the BT-COF treated under the different conditions: (a) dispersed in 0.1 M ascorbic acid solution for 30 min without irradiation (black), (b) irradiated (λ > 300 nm) for 30 min in water (red), and (c) dispersed in 0.1M ascorbic acid solution for photoreduction of $HPtCl_6$ to Pt nanoparticles under irradiation (λ > 300 nm) for 30 min (green).

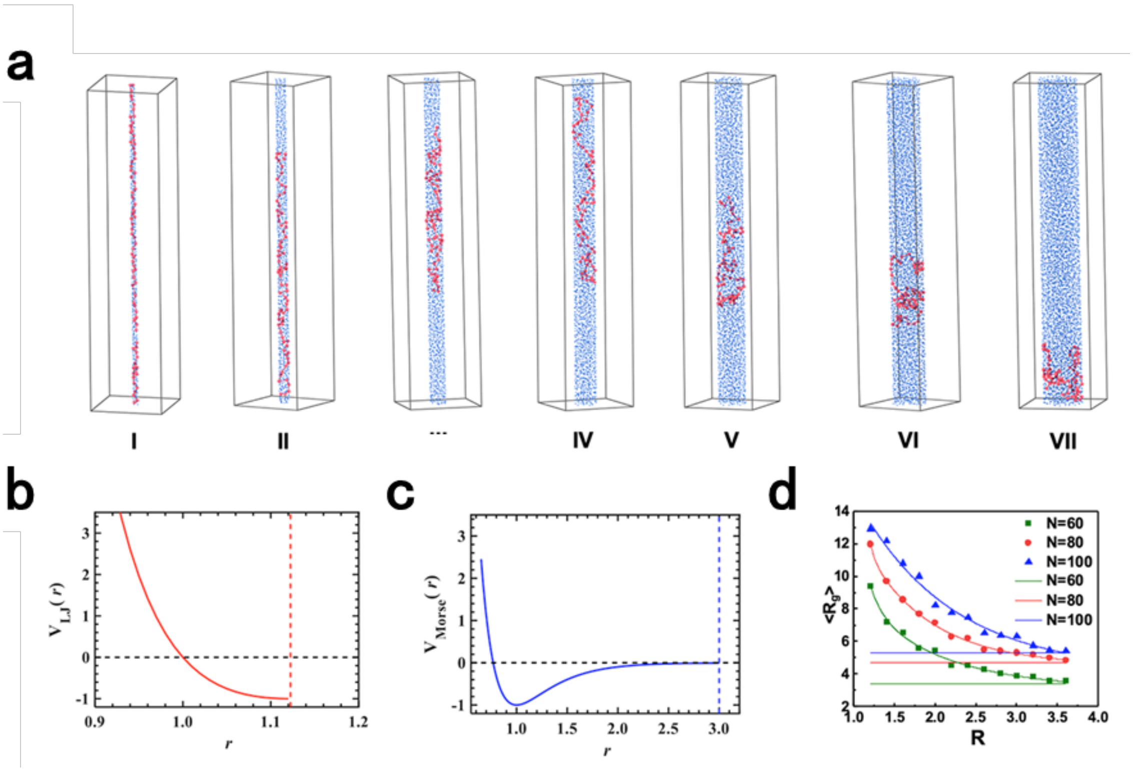


**Fig. 42** (a) DPD simulation for the conformations of PEG chains (the linked 80 red beads) and water solvents (single blue beads) in a variety of nanotubes with the radius of 1.2 (I), 1.6 (II), 2.0 (III), 2.4 (IV), 2.8 (V), 3.2 (VI), and 3.6 (VII), respectively. (b) The distance r *vs.* Lennard-Jones repulsion interaction between nanotube wall and PEG-chain model. (c) The distance r *vs.* H-bonding attraction interaction between nanotube wall and PEG-chain model. (d) The radius of gyration *vs.* the radius of the nanotube. The fitted curves represent the cylinder constraint state, the parallel straight lines represent the bulk solvent state, and N represents the number of repeated beads in the polymeric chain.

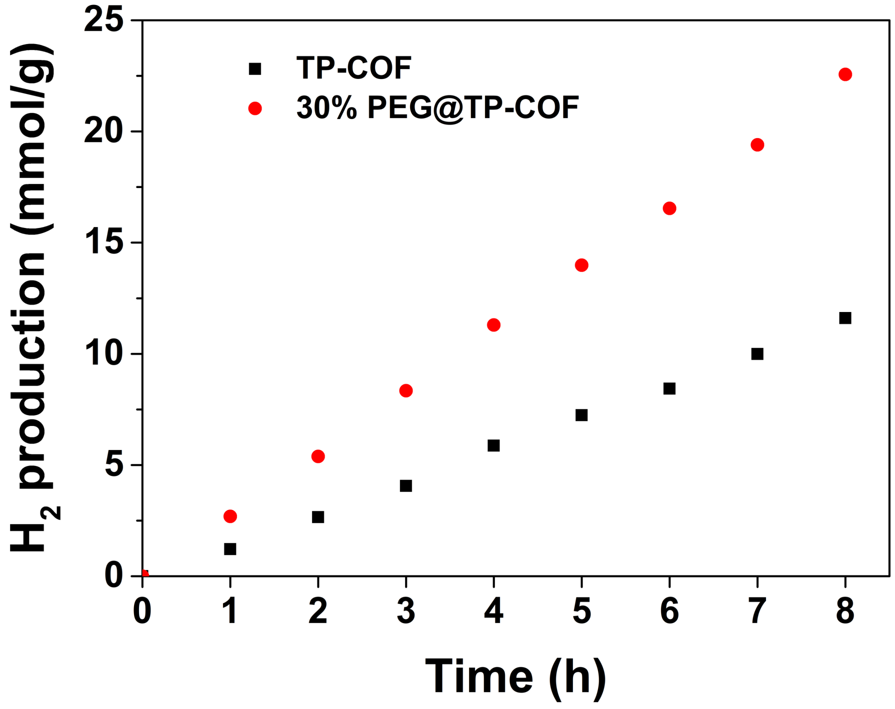


**Fig. 43** Time course of $H_2$ evolution using TP-COF and 30%PEG@TP-COF.

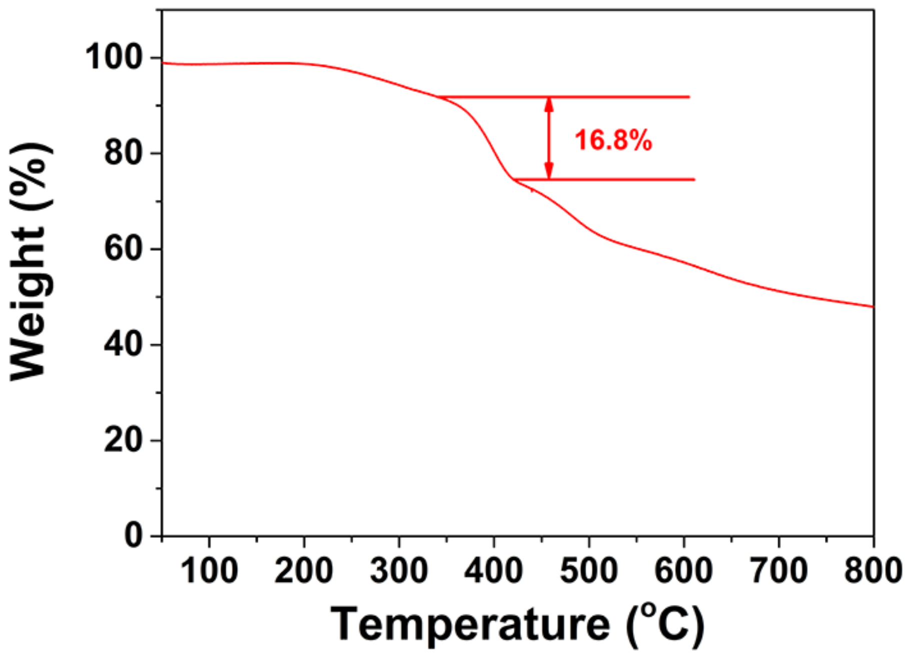


**Fig. 44** TGA curve of the 30%PEG@BT-COF recycled after 48-h photocatalytic test. The weight loss of the remaining PEG in the recycled photocatalyst is found to be 16.8wt.%. Compared to the initial PEG@BT-COF containing 22.7wt.% of PEG, there is 5.9 wt.% of PEG leaking out from the composite PEG@BT-COF during the 48-h photocatalysis test.

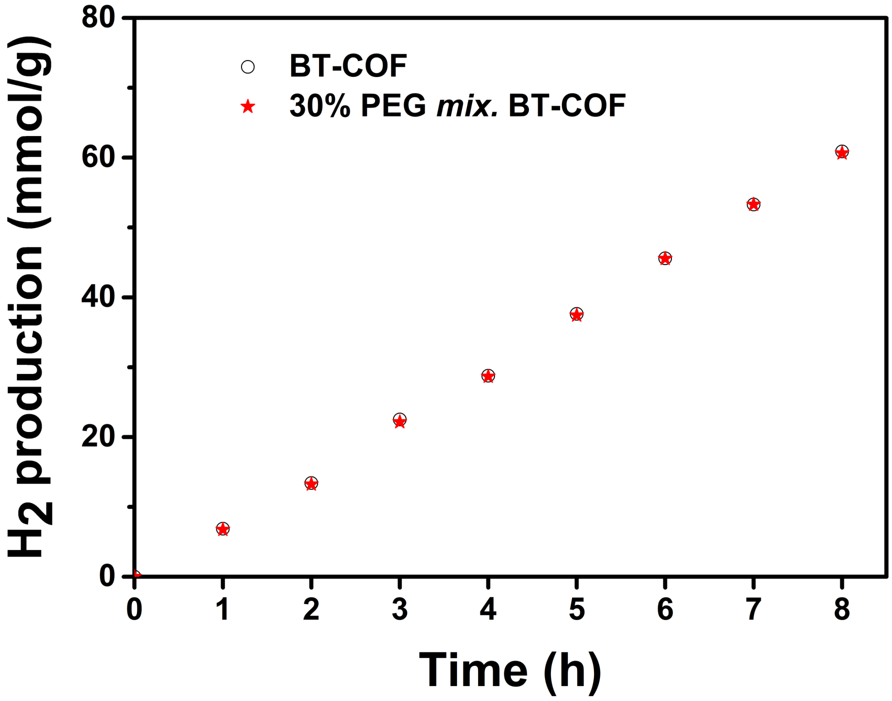


**Fig. 45** Time course of $H_2$ evolution using 30%PEG mix. BT-COF and BT-COF, respectively.

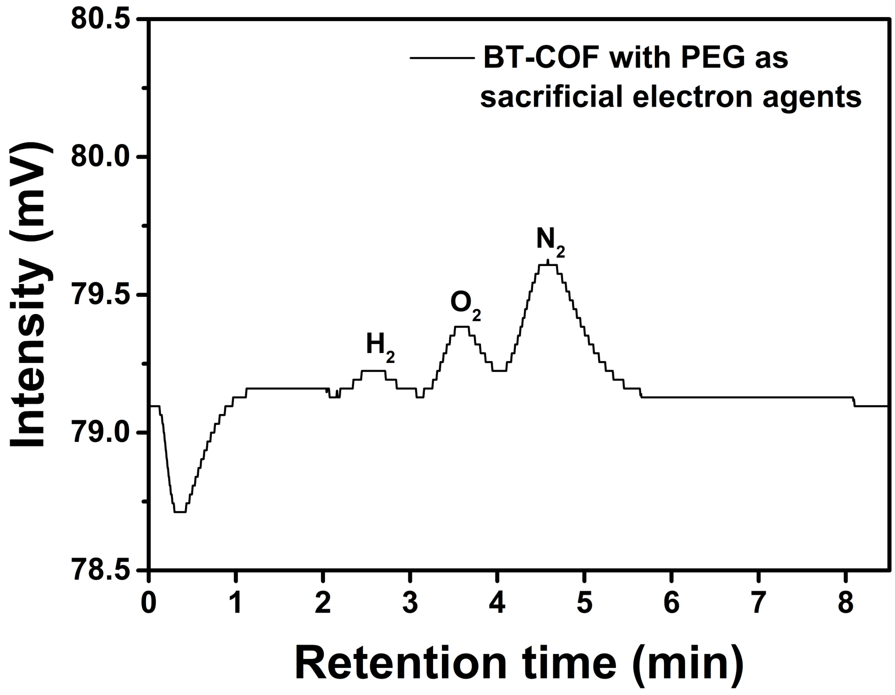


**Fig. 46** GC profile of evolved $H_2$ in the photocatalytic reaction, using BT-COF (10 mg) with 5wt% Pt as a co-catalyst and 30wt% PEG ($M_w$ = 20kDa) as a sacrificial electron donor. There is negligible $H_2$ produced in the system under 4-h visible irradiation (λ>420 nm).

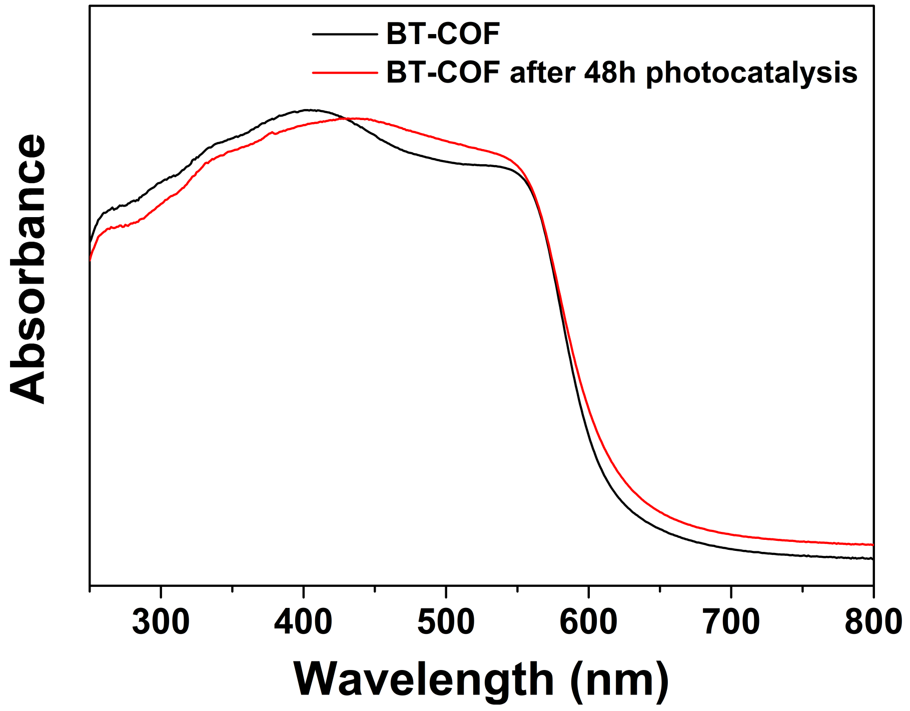


**Fig. 47** UV-vis diffuse reflectance spectra of the BT-COF before (black) and after (red) the 48-h photocatalytic test. The characterization was performed after 48 h $H_2$ evolution experiment under visible light ($\lambda > 420$ nm).

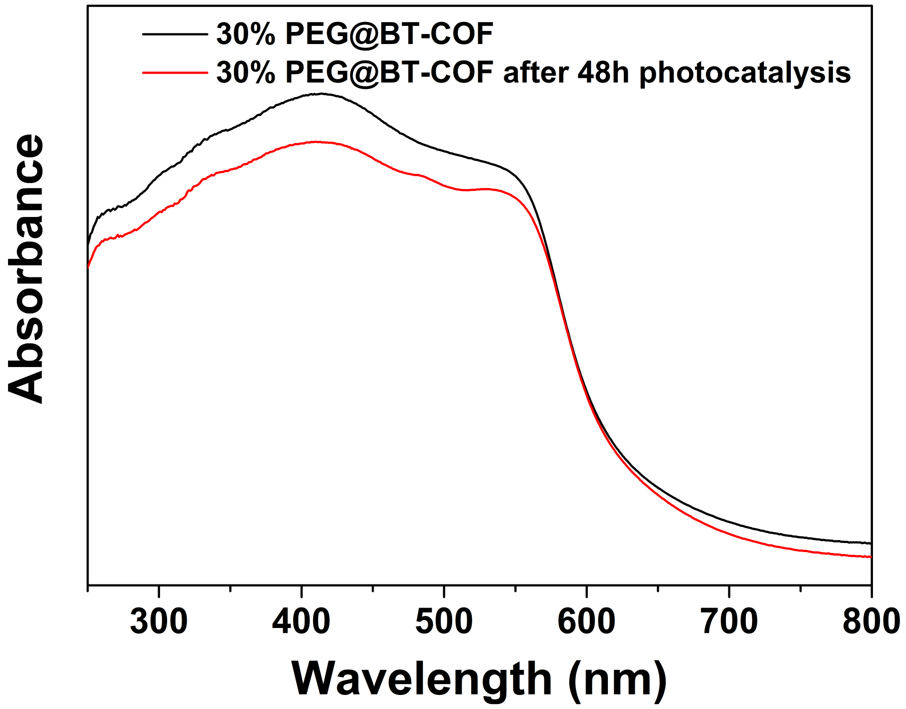


**Fig. 48** UV-vis diffuse reflectance spectra of the 30%PEG@BT-COF before (black) and after (red) the 48-h photocatalytic test (λ > 420 nm).

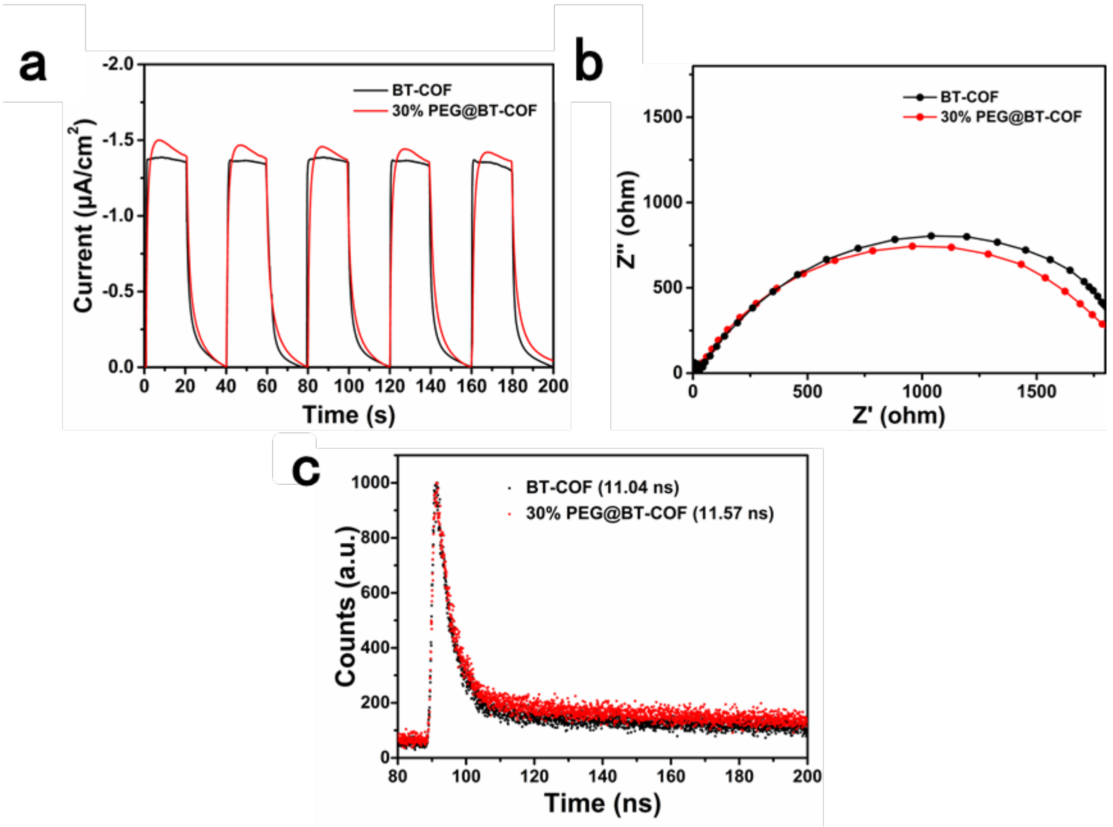


**Fig. 49** Transient photocurrent responses (a), Nyquist plots (b) and TCSPC measured fluorescence decay profiles (c) of BT-COF and 30%PEG@BT-COF, respectively. both samples were measured without Pt cocatalyst prior to photocatalytic test.

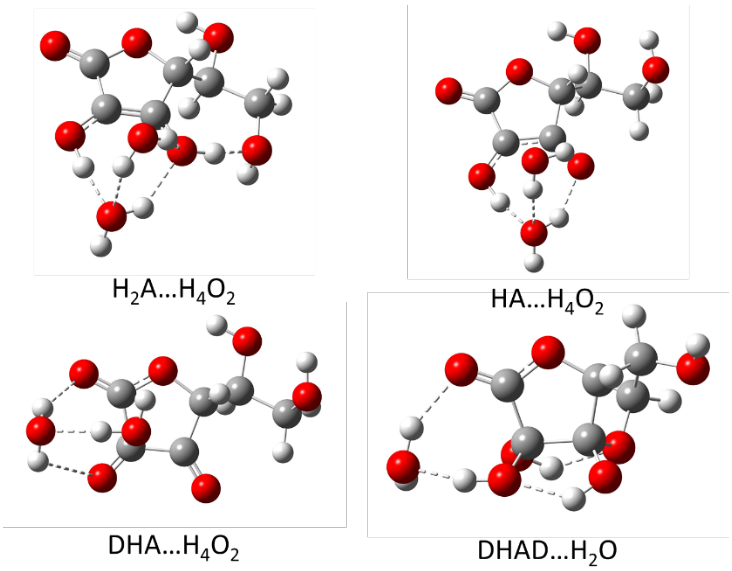


**Fig. 50** Schematic geometries of ascorbic acid with two water molecules ($H_2A…H_4O_2$), the cluster of ascorbate radical with two water molecules ($HA…H_4O_2$), dehydroascorbic acid with two water molecules ($DHA…H_4O_2$), and the bicyclic diol form of DHA with one water molecule ($DHAD…H_2O$).

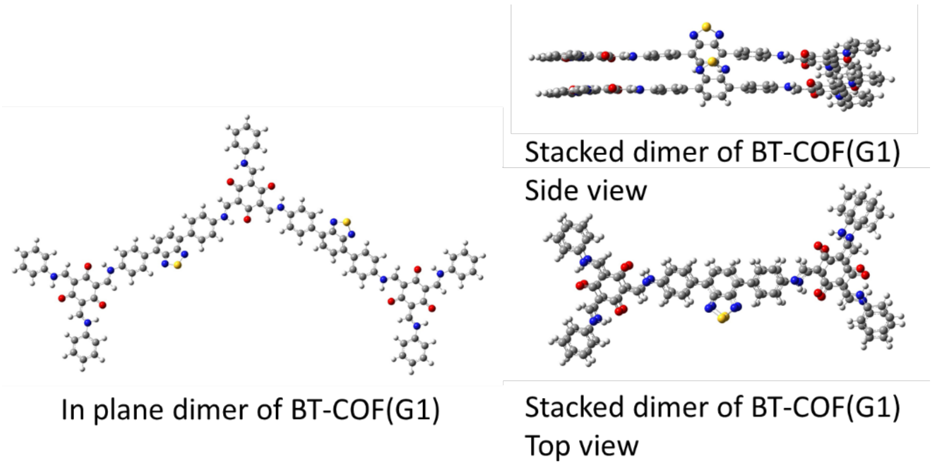


**Fig. 51** Schematic geometry of the in plane and stacked dimer model of BT-COF(G1)

**Table 8.** Electron coupling $J_{ij}$, the spectral overlap, and an estimate of the electron transfer rate for G1 BT-COF and TP-COF calculated using TD-CAMB3LYP/def2SVP. Values in parenthesis is obtained from prefect overlapping stacked dimer calculations.

| | $J_{ij}$ (eV) | Overlap ($eV^{-1}$) | $k_{ET}$ ($s^{-1}$) |
|---|---|---|---|
| BT-COF (out of plane) | 0.058<br>(0.11) | 0.019 | $1.3\times10^{12}$ |
| BT-COF (in plane) | 0.033 | 0.019 | $2.0\times10^{11}$ |
| TP-COF (out of plane) | 0.056 | 0.266 | $1.8\times10^{13}$ |
| TP-COF (in plane) | 0.047 | 0.266 | $5.6\times10^{12}$ |

**Supplementary References**


1. Lan, Y. et al. Materials genomics methods for high-throughput construction of COFs and targeted synthesis. *Nat. Commun.* **9**, 5274 (2018).
2. Mayo, S. L., Olafson, B. D. & Goddard W. A. DREIDING: a generic force field for molecular simulations. *J. Phys. Chem.* **94**, 8897−8909 (1990).
3. Rappe, A. K. & Goddard W. A. Charge equilibration for molecular dynamics simulations. *J. Phys. Chem.* **95**, 3358−3363 (1991).
4. Elstner, M. et al. Self-consistent-charge density-functional tight-binding method for simulations of complex materials properties. *Phys. Rev. B.* **58**, 7260−7268 (1998).
5. Niehaus, T. A., Elstner, M., Frauenheim, Th. & Suhai, S. Application of an approximate density-functional method to sulfur containing compounds. *J. Mol. Struct.: THEOCHEM.* **541**, 185−194 (2001).
6. Kresse, G. & Furthmuller, J. Efficient iterative schemes for ab initio total-energy calculations using a plane-wave basis set. *Phys. Rev. B: Condens. Matter Mater. Phys.* **54**, 11169−11186 (1996).
7. Yanai, T., Tew, D. P. & Handy, N. C. A new hybrid exchange-correlation functional using the Coulomb-attenuating method (CAM-B3LYP). *Chem. Phys. Lett.* **393**, 51−57 (2004).
8. Weigend, F. Accurate Coulomb-fitting basis sets for H to Rn. *Phys. Chem. Chem. Phys.* **8**, 1057−1065 (2006).
9. Casida, M. E., Jamorski, C., Casida, K. C. & Salahub, D. R. Molecular excitation energies to high-lying bound states from time-dependent density-functional response theory: Characterization and correction of the time-dependent local density approximation ionization threshold. *J. Chem. Phys.* **108**, 4439−4449 (1998).
10. Stratmann, R. E., Scuseria, G. E. & Frisch, M. J. An efficient implementation of time-dependent density-functional theory for the calculation of excitation energies of large molecules. *J. Chem. Phys.* **109**, 8218−8224 (1998).

11. Marenich, A. V., Cramer, C. J. & Truhlar, D. G. Universal solvation model based on solute electron density and a continuum model of the solvent defined by the bulk dielectric constant and atomic surface tensions. *J. Phys. Chem. B*. **113**, 6378–6396 (2009).

12. Bahers, T. L., Adamo, C. & Ciofini, I. A qualitative index of spatial extent in charge-transfer excitations. *J. Chem. Theory Comput.* **7**, 2498–2506 (2011).

13. Grimme, S., Ehrlich, S. & Goerigk, L. Effect of the damping function in dispersion corrected density functional theory. *J. Comp. Chem*. **32**, 1456–1465 (2011).

14. Guiglion, P., Butchosa, C. & Zwijnenburg, M. A. Polymeric watersplitting photocatalysts; a computational perspective on the water oxidation conundrum. *J. Mater. Chem. A.* **2**, 11996–12004 (2014).

15. Guiglion, P., Butchosa, C. & Zwijnenburg, M. A. Polymer photocatalysts for water splitting: insights from computational modeling. *Macromol. Chem. Phys.* **217**, 344−353 (2016).

16. Tu, Y. J., Njus, D. & Schlegel, H. B. A theoretical study of ascorbic acid oxidation and HOO·/$O_2{\cdot}^-$ radical scavenging. *Org. Biomol. Chem.* **15,** 4417–4431 (2017).

17. Atkins, P. & Friedman, R. Molecular Quantum Mechanics 4$^{th}$ edition, Oxford University Press, New York (2005).

18. Son, H. et al. Light-harvesting and ultrafast energy migration in porphyrin-based metal−organic frameworks. *J. Am. Chem. Soc.* **135**, 862−869 (2013).

19. Zhang, Q. et al. Forster energy transport in metal−organic frameworks is beyond step-by-step hopping *J. Am. Chem. Soc.* **138**, 5308−5315 (2016).

20. Guo, J. et al. Conjugated organic framework with three-dimensionally ordered stable structure and delocalized π clouds. *Nat. Commun*. 4, 2736 (2013).

21. Wang, X. et al. Sulfone-containing covalent organic frameworks for photocatalytic hydrogen evolution from water. *Nat. Chem.* **10**, 1180–1189 (2018).

22. Flanders, N. C. et al. Large exciton diffusion coefficients in two-dimensional covalent organic frameworks with different domain sizes revealed by ultrafast exciton dynamics. *J. Am. Chem. Soc.* **142**, 14957−14965 (2020).
23. Jakowetz, A. C. et al. Excited-state dynamics in fully conjugated 2D covalent organic frameworks. *J. Am. Chem. Soc.* **141**, 11565−11571 (2019).
24. Hoogerbrugge, P. J. & Koelman, J. M. V. A. Simulating microscopic hydrodynamic phenomena with dissipative particle dynamics. *Europhys. Lett.* **19**, 155–160 (1992).
25. Groot, R. D. & Warren, P. B. Dissipative particle dynamics: Bridging the gap between atomistic and mesoscopic simulation. *J. Chem. Phys.* **107**, 4423–4435 (1997).
26. Zhu, Y. L. et al. GALAMOST: GPU-accelerated large-scale molecular simulation toolkit. *J. Comput. Chem.* **34**, 2197–2211 (2013).
27. Phillips, C. L., Anderson, J. A. & Glotzer, S. C. Pseudo-random number generation for Brownian Dynamics and Dissipative Particle Dynamics simulations on GPU devices. *J. Comput. Phys.* **230**, 7191–7201 (2011).
28. Stegbauer, L., Schwinghammer, K. & Lotsch, B. V. A hydrazone-based covalent organic framework for photocatalytic hydrogen production. *Chem. Sci.* **5**, 2789–2793 (2014).
29. Vyas, V. S. et al. A tunable azine covalent organic framework platform for visible light-induced hydrogen generation. *Nat. Commun.* **6**, 8508 (2016).
30. Banerjee, T. et al. Single-site photocatalytic $H_2$ evolution from covalent organic frameworks with molecular cobaloxime cocatalysts. *J. Am. Chem. Soc.* **139**, 16228–16234 (2017).
31. Haase, F., Banerjee, T., Savasci, G., Ochsenfeld, C. & Lotsch, B.V. Structure-property-activity relationships in a pyridine containing azine-linked covalent organic framework for photocatalytic hydrogen evolution. *Faraday Discuss.* **201**, 247–264 (2017).

32. Pachfule, P. et al. Diacetylene functionalized covalent organic framework (COF) for photocatalytic hydrogen generation. *J. Am. Chem. Soc.* **140**, 1423–1427 (2018).
33. Stegbauer, L. et al. Photocatalysis: tailor-made photoconductive pyrene-based covalent organic frameworks for visible-light driven hydrogen generation. *Adv. Energy Mater.* **8**, 1870107 (2018).
34. Liu, M. et al. Crystalline covalent triazine frameworks by in situ oxidation of alcohols to aldehyde monomers. *Angew. Chem. Int. Ed.* **57**, 11968–11972 (2018).
35. Jin, E. et al. 2D sp2 carbon-conjugated covalent organic frameworks for photocatalytic hydrogen production from water. *Chem* **5**, 1–16 (2019).
36. Bi, S. et al. Two-dimensional semiconducting covalent organic frameworks via condensation at arylmethyl carbon atoms. *Nat. Commun.* **10**, 2467 (2019).
37. Ming, J. et al. Hot π-electron tunneling of metal-insulator-COF nanostructures for efficient hydrogen production. *Angew. Chem. Int. Ed.* **58**, 18290–18294 (2019).
38. Zhang, S. et al. Strong base assisted synthesis of crystalline covalent triazine framework with high hydrophilicity via benzylamine monomer for photocatalytic water splitting. *Angew. Chem. Int. Ed.* **59**, 6007–6014 (2020).
